# Small sample sizes: A big data problem in high-dimensional data analysis

**DOI:** 10.1177/0962280220970228

**Published:** 2020-11-24

**Authors:** Frank Konietschke, Karima Schwab, Markus Pauly

**Affiliations:** 1 Charité-Universitätsmedizin Berlin, Humboldt-Universität zu Berlin, and Berlin Institute of Health, Institute of Biometry and Clinical Epidemiology, Charitéplatz 1, Berlin, Germany; 2 Berlin Institute of Health (BIH), Anna-Louisa-Karsch-Straße 2, Berlin, Germany; 3 Institute of Pharmacology, Charité-Universitätsmedizin Berlin, Charitéplatz 1, Berlin, Germany; 4 Department of Statistics, TU Dortmund University, Dortmund, Germany

**Keywords:** Multiple contrast tests, max *t*-test, repeated measures, resampling, simultaneous confidence intervals

## Abstract

In many experiments and especially in translational and preclinical research, sample sizes are (very) small. In addition, data designs are often high dimensional, i.e. more dependent than independent replications of the trial are observed. The present paper discusses the applicability of *max t*-test-type statistics (multiple contrast tests) in high-dimensional designs (repeated measures or multivariate) with small sample sizes. A randomization-based approach is developed to approximate the distribution of the maximum statistic. Extensive simulation studies confirm that the new method is particularly suitable for analyzing data sets with small sample sizes. A real data set illustrates the application of the methods.

## 1 Introduction

Small sample sizes occur in various research experiments and especially in preclinical (animal) studies due to ethical, financial, and general feasibility reasons. Such studies are essential and an important part of translational medicine and other areas (e.g. rare diseases). Often, less than 20 animals per group are involved, and thus making valid inferences in these studies becomes a challenging part. In addition to the small sample sizes, repeated measurements as well as multiple endpoints are often observed on the experimental units (animals), naturally leading to a “large p, small n” situation and thus to a high-dimensional data design. Note that high-dimensional data do not only occur in animal studies, medical imaging and genomics are other well-known application areas. The first statistical problem at hand is neither the high dimensionality of the data nor the relatively low statistical power of the tests when sample sizes are very small—it is the accurate type-1 error rate control of the methods. Many of the existing statistical methods require moderate or large sample sizes and therefore tend to not control the type-1 error rate properly when sample sizes are very small; they either behave liberal and over-reject the null hypothesis or are conservative. Exact techniques (i.e. procedures that rely on the exact distribution of a test statistic for any finite sample size *n*) would be a great choice, but they typically rely on strict model assumptions that can hardly be verified—at least in more complex models. Indeed, making any assumptions about the underlying distributions (e.g. based on boxplots), verifying equality of variances, specific covariance structures, etc. is quasi impossible when sample sizes are so small and thus, methods, which do not rely on strict model assumptions, are the methods of choice. All in all, besides the often discussed problem of high dimensionality, small sample sizes increase the challenge of a robust and especially accurate data analysis in such situations.

Beyond these challenges and even though the statistical designs themselves are usually complex, the research questions and study aims are often very specific. These may be tackled by applying global testing procedures, which have been developed for different high-dimensional repeated measures and multivariate ANOVA models by several authors.^1–8^ Furthermore, multivariate tests based on interpoint distances have been proposed.^9–12^ Testing global null hypotheses and herewith answering the question whether any difference among the repeated measurements per or across endpoints exists, however, does usually not answer the main question of the practitioners—that is the specific localization of the responsible experimental conditions that lead to the overall significance conclusion. A modern data analysis requires the use of multiple comparison procedures that control the family-wise error rate in the strong sense and that are flexible in the way that they can be used to test arbitrary global and local null hypotheses and lead to *compatible* simultaneous confidence intervals (SCIs) for the underlying treatment effects. Furthermore, due to the often complex dependency structure across the repeated measurements and contrasts, the multiple comparison method should take the correlations of the different tests statistics for powerful data analysis into account. Such methods are also known as *multiple contrast tests* (MCTP) and are based on the maximum value of a vector of possibly correlated *t*-test type statistics (max *t*-test statistic). Computing its exact distribution without making strict distributional assumptions is impossible in general designs.^13^ Therefore, approximations of its asymptotic distribution are needed for making inferences. Recently, it has been suggested^14^ to estimate the distribution of the maximum value within a bootstrap simulation-based framework using the empirical correlation matrix. Simulation studies indicate, however, that sample sizes $n_{i}\geq 50$ are necessary for an accurate type-1 error rate control. When sample sizes are smaller, the methods tend to be liberal (see Section 5). In the present paper, a modification of the proposed method will be introduced that does not require the estimation of the correlation matrix. Extensive numerical studies show that the new approach controls the type-1 error very accurately even when sample sizes are very small and data do not follow multivariate normal distributions with equal covariance matrices.

The paper is organized as follows. In Section 2, a high-dimensional preclinical study on Azheimer’s disease with small sample sizes is described. Existing methodology for the statistical evaluation of such designs is explained in Section 3. Here, its behavior in small sample size situations is also investigated, which motivates the development of a different approximation of the distribution of the max *t*-test in Section 4. The qualities of the competing approximations are compared in extensive simulation studies in Section 5. The paper closes with the evaluation of the example and a discussion about the results in Sections 6 and 7, respectively. Theoretical properties of the new approximation and proofs are provided in the supplementary material file. Throughout the paper, $I_{d}$ denotes the *d*-dimensional unit matrix, $J_{d}=1_{d}1\prime_{d}$ the *d *×* d* matrix of 1 s, where $1_{d}=(1,\ldots ,1)\prime_{d\times 1}$.

## 2 A motivating example

**Figure 1.**
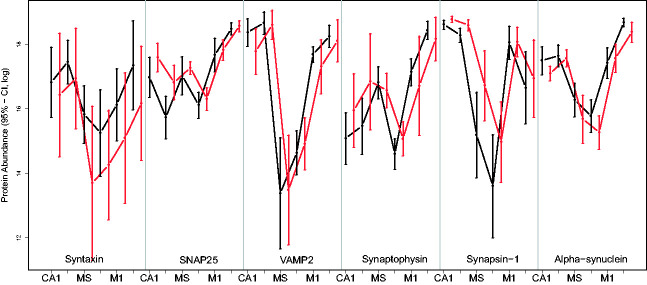
Confidence interval plot (95%) for each protein× region× group combination in the protein abundance trial. Each confidence interval has been computed by inverting the corresponding one-sample *t*-test statistic using 97.5%-t-quantiles from a *t*-distribution with $n_{i}-1$ degrees of freedom.

As a motivating example, we consider a part of a preclinical study on Alzheimer's disease conducted in the Institute of Pharmacology at the Charité university medical center in Berlin, Germany. The study involves $n_{1}=10$ wild-type mice (group 1) and $n_{2}=9$ L1 tau-transgenic type (group 2) mice. As usual, the sample sizes of this preclinical research trial are pretty small. The abundance of each of the six different proteins Syntaxin, SNAP25, VAMP2, Synaptophysin, Synapsin-1 and Alpha-synuclein were measured in six different regions of the brain of every mouse. The regions of interest were pre-defined as hippocampal CA1 region (CA1), visual cortex (VC), medial septum (MS), vertical limb of the diagonal band of Broca (VDB), primary motor cortex (M1) and nucleus accumbens (ACB), respectively. Thus, 36 observations were made on every mouse, while the number of dependent replicates exceeds the number of independent replications of the trial. Therefore, the statistical design represents a classical “large p, small n” situation with small sample sizes. We note that generating such data requires very advanced technology. For graphical representation and easy display of the data, the protein abundances were log-transformed. The results are displayed in confidence interval plots (with chosen local level 95%) in Figure 1. For illustration, additional dotplots and boxplots of the data are displayed in the supplementary material file.

The dotplots give the impression that the protein abundances are roughly symmetrically distributed. However, since sample sizes are so small, making any assumption about the underlying distribution would be questionable. It can also be seen that few “outliers” are present. These values have been kept in the data set, because the range of protein abundance measurements is usually very wide. Therefore, these values are not outliers in the classical sense and provide useful information about the protein levels in the respective brain regions. Furthermore, the confidence intervals displayed in Figure 1 show a fairly amount of variance heteroscedasticity. Therefore, the data of this trial can be modeled by independent and identically distributed random vectors

(1) $$X_{ik}=(X_{i1k},\ldots ,X_{\mathrm{idk}})\prime ∼F_{i},\;i=1,2;\;k=1,\ldots ,n_{i};\;N=n_{1}+n_{2}$$

with expectation $E(X_{i1})=\mu_{i}=(\mu_{i1},\ldots ,\mu_{id})\prime$ and covariance matrix $\mathrm{Cov}(X_{\text{i1}})=\Sigma_{\text{i}}>\text{0}$, *i *=* *1, 2. For a convenient notation, the index s=1,…,d represents the repeated measures in the regions under each of the different endpoints. Here, we set

$$d:\{\begin{array}{l} \text{Dimension}\text{Protein}\text{Region}\\ 1\leq d\leq 6\text{Syntaxin}\text{(CA1, VC, MS, VDB, M1, ACB)}\\ 7\leq d\leq 12\text{SNAP25}\text{(CA1, VC, MS, VDB, M1, ACB)}\\ 13\leq d\leq 18\text{VAMP2}\text{(CA1, VC, MS, VDB, M1, ACB)}\\ 19\leq d\leq 24\text{Synaptophysin}\text{(CA1, VC, MS, VDB, M1, ACB)}\\ 25\leq d\leq 30\text{Synapsin-1}\text{(CA1, VC, MS, VDB, M1, ACB)}\\ 31\leq d\leq 36\text{Alpha-synuclein}\text{(CA1, VC, MS, VDB, M1, ACB)} \end{array}$$

and arrange all analyses according to this order. In general, data modeled by Equation (1) can either be repeated measures (measurements on the same scale), multivariate data (measurements on different scales) or combinations thereof. For a convenient notation of the hypotheses, let $\mu =(\mu_{1}\prime ,\mu_{2}\prime )\prime$ denote the combined vector of the expectations in both groups. Besides the questions whether abundances of the proteins in the different regions of the brain differ, the study specifically aims to locate specific group × region interactions for each of the proteins. From a medical point of view, these would expose the proteins and especially the regions of the brain as biomedical biomarkers for Alzheimer’s disease. To be more specific, let $\mu_{ij}^{\left(P\right)}$ denote the expected protein abundance in group *i* under region *j* of protein *P*, where *i *=* *1, 2; *j* ∈{CA1, VC, MS, VDB, M1, ACB} and P∈P={Syntaxin, SNAP25, … , Alpha-synuclein}, respectively. For each single protein, the major aim is to (i) decide whether there is a group × region interaction and if so (ii) where. This can be achieved by simultaneously testing whether the group-wise differences $\mu_{1j}^{\left(P\right)}-\mu_{2j}^{\left(P\right)}$ are identical for all regions j=1,…,6 for each protein P=1,…,6. This leads to testing the family of 72 multiple null hypotheses

$$\Omega =\left\{H_{0}^{\left(j,P\right)}:\mu_{ij}^{\left(P\right)}={\bar{\mu }}_{i\cdot }^{\left(P\right)}+{\bar{\mu }}_{\cdot j}^{\left(P\right)}-{\bar{\mu }}_{\cdot \cdot }^{\left(P\right)},i=1,2,j=1,\ldots ,6,P\in P\right\}$$

at multiple level *α*. Here,

$${\bar{\mu }}_{i\cdot }^{\left(P\right)}=\frac{1}{6}\sum_{j=1}^{6}\mu_{ij}^{\left(P\right)},\;{\bar{\mu }}_{\cdot j}^{\left(P\right)}=\frac{1}{2}\sum_{i=1}^{2}\mu_{ij}^{\left(P\right)}\;\text{ and }\;{\bar{\mu }}_{\cdot \cdot }^{\left(P\right)}=\frac{1}{12}\sum_{i=1}^{2}\sum_{j=1}^{6}\mu_{ij}^{\left(P\right)}$$

denote the corresponding means of expectations as known from linear model theory, where i=1,2;j=1,…,6;P∈P. Thus, the hypotheses are nothing but testing whether the differences of the expectations $\mu_{1j}^{\left(P\right)}-\mu_{2j}^{\left(P\right)},j=1,\ldots ,6$ are identical for each protein. For simplicity, we rewrite the above using matrix notation and equivalently obtain

$$\Omega =\{H_{0}^{\left(\ell \right)}:c_{\ell }\prime \mu =0,\ell =1,\ldots ,q\}$$

where $c_{\ell }\prime$ denotes the ℓth row vector of the contrast matrix as used in

(2) $$H_{0}^{\mu }:C\mu =0,\;\text{ with }\;\;C=\left(\underset{P\in P}{⊕}P_{6}\right)\left(I_{36}\vdots -I_{36}\right)$$

Here, in Equation (2), we denote with $P_{m}=I_{m}-\frac{1}{m}1_{m}1_{m}\prime$ the *m*-dimensional centering matrix, while ⊕ describes the direct sum to build a block diagonal matrix.

Note that this summarizing matrix notation enables us to describe the 72 null hypotheses equivalently by only q = 36 which shall be tested using $n_{1}=10$ and $n_{2}=9$ independent replications. Therefore, the above is a high-dimensional multiple testing problem. An existing statistical method to analyze the data will be discussed in the next section.

## 3 Existing methodology

The high dimensionality of the testing problem considered here makes the data analysis complex in the sense that the computation of the critical values for making statistical inference becomes an issue. Recently, Chang et al.^14^ propose a simulation-based inference method for high-dimensional data. The procedure is valid for large dimensions and sample sizes. The case of small sample sizes has not been considered and therefore its applicability in such situations intrigues a detailed investigation. In their original paper, both the cases of studentized and non-studentized statistics have been considered. For the ease of read, we will concentrate on the studentized statistics in the following only. By doing so, we follow the guidelines of resampling studentized statistics.^15–18^ First, we will rewrite the null hypothesis and introduce the statistics in the same way as they were described by Chang et al.^14^ who propose to test the equality of expectations of the *q*-variate random vectors $Y_{ik}=(Y_{i1k},\ldots ,Y_{\mathrm{iqk}})\prime$ by $H_{0}^{\theta }:\theta_{1}=\theta_{2}$. These considerations show that the two statistical testing problems are identical. However, we will propose a different way of estimating the critical values for making reliable inference later in Section 4.

Note that the null hypothesis $H_{0}:C\mu =0$ as given in Equation (2) can be equivalently written as the “standard” multivariate null hypothesis

$$H_{0}^{\theta }:\theta_{1}=\theta_{2}$$

where $\theta_{i}=(\theta_{i1},\ldots ,\theta_{iq})\prime =E(Y_{ik})$ denotes the expectation of the transformed vectors $Y_{ik}\prime =X_{ik}\prime \left({⊕}_{s=1}^{6}P_{6}\right)$. In order to test $H_{0}^{\theta }$ against $H_{1}^{\theta }:\theta_{1}\neq \theta_{2}$, consider the maximum of the *q* component-wise *t*-test type statistics

(3) $$T_{0}=\max\{|T_{1}|,\ldots ,|T_{q}|\},\;\text{ where }\;T_{\ell }=\sqrt{N}\frac{\left({\bar{Y}}_{1\ell \cdot }-{\bar{Y}}_{2\ell \cdot })-(\theta_{1\ell }-\theta_{2\ell }\right)}{\sqrt{{\hat{v}}_{1,\ell \ell }/n_{1}+{\hat{v}}_{2,\ell \ell }/n_{2}}}$$

denotes the studentized difference of the means ${\bar{Y}}_{i\ell \cdot }=\frac{1}{n_{i}}\sum_{k=1}^{n_{i}}Y_{i\ell k}$ with the empirical variances ${\hat{v}}_{i,\ell \ell }=N{\left(n_{i}-1\right)}^{-1}\sum_{k=1}^{n_{i}}{\left(Y_{i\ell k}-{\bar{Y}}_{i\ell \cdot }\right)}^{2},\;i=1,2;\;\ell =1,\ldots ,q$. The use of maximum *t*-statistics plays an important role in preclinical research, because local test decisions can be made using adjusted *p*-values for the comparisons $H_{0}^{\left(\ell \right)}:\theta_{1\ell }=\theta_{2\ell },\;\ell =1,\ldots ,q$. Second, each *t*-statistic describes the distance of the observed mean difference to its respective null hypothesis in units of standard deviations. However, for the computation of the local p-values, the distribution of *T*_0_ must be known, at least approximately. Suppose for a moment that it is known, then the individual null hypothesis $H_{0}^{\left(\ell \right)}:\theta_{1\ell }=\theta_{2\ell }$ will be rejected at multiple level *α*, if

(4) $$\left|T_{\ell }|\geq z_{1-\alpha }(\max\right)$$

where $z_{1-\alpha }(\max)$ denotes the (1−α)-quantile from the distribution of *T*_0_. Compatible SCIs for the effects $\delta_{\ell }=\theta_{1\ell }-\theta_{2\ell }$ are given by

(5) $$CI_{\ell }=\left[{\bar{Y}}_{1\ell \cdot }-{\bar{Y}}_{2\ell \cdot }\mp \frac{z_{1-\alpha }(\max)}{\sqrt{N}}\sqrt{{\hat{v}}_{1,\ell \ell }/n_{1}+{\hat{v}}_{2,\ell \ell }/n_{2}}\right]$$

Finally, the global null hypothesis $H_{0}:C\mu =0$ will be rejected, if

(6) $$T_{0}\geq z_{1-\alpha }(\max)$$

In such general models (even under the assumption of multivariate normality), however, the exact distribution of *T*_0_ remains unknown^19^ and approximate methods are needed for estimating the distribution of *T*_0_. In low-dimensional designs (fixed dimension *d* and contrasts *q*), the vector of *t*-statistics

(7) $$T=(T_{1},\ldots ,T_{q})\prime$$

follows, asymptotically, as N→∞, a multivariate normal distribution with expectation 0 and correlation matrix

(8) $$R=D^{-1/2}VD^{-1/2},\;\text{ where }$$

(9) $$V=\mathrm{Cov}(\sqrt{\text{N}}({\bar{Y}}_{\text{1}\cdot }\text{-}{\bar{Y}}_{\text{2}\cdot }))\text{=N}\left(\ddot{C}\Sigma_{\text{1}}\ddot{C}\prime {\text{/n}}_{\text{1}}\text{+}\ddot{C}\Sigma_{\text{2}}\ddot{C}\prime {\text{/n}}_{\text{2}}\right)$$

with

$$\ddot{C}=\overset{6}{\underset{s=1}{⊕}}P_{6}$$

denotes the covariance matrix of the differences in means and D denotes the diagonal matrix obtained from the diagonal elements of V. These considerations show that the (asymptotic) joint distribution of the vector of *t*-statistics T (and therefore of *T*_0_) depends on the unknown correlation matrix and is non-pivotal. This is intuitively clear, since the higher the statistics are correlated, the smaller should be the critical value $z_{1-\alpha }(\max)$. Indeed, in case of a perfect correlation, the above reduces to a univariate testing problem. Anyway, the correlation matrix is unknown and the above cannot be used for making inferences in its present form. Chang et al.^14^ propose to first estimate the correlation matrix by its empirical counterpart

$$\hat{R}={\hat{D}}^{-1/2}\hat{V}{\hat{D}}^{-1/2},\;\text{ where }$$

$$\hat{V}=N\left({\hat{V}}_{1}/n_{1}+{\hat{V}}_{2}/n_{2}\right),\text{ and }$$

$${\hat{V}}_{i}=\frac{1}{n_{i}-1}\sum_{k=1}^{n_{i}}\left(Y_{ik}-{\bar{Y}}_{i\cdot })(Y_{ik}-{\bar{Y}}_{i\cdot }\right)\prime ,\;i=1,2$$

denote the empirical covariance matrix of the random vectors $Y_{ik}$. Analogously, $\hat{D}$ denotes the diagonal matrix obtained from the diagonal elements of $\hat{V}$. Next, they propose to generate *M* random vectors

(10) $$Y_{b}^{*}∼N(0,\hat{R}),\;b=1,\ldots ,M$$

from a multivariate normal distribution with expectation 0 and correlation matrix $\hat{R}$ and to estimate the critical value $z_{1-\alpha }(\max)$ by computing the (1−α)-quantile $y_{1-\alpha }^{*}(\max)$ of the values $Y_{0,1}^{*},\ldots ,Y_{0,M}^{*}$, where $Y_{0,b}^{*}=\max\{|Y_{b1}^{*}|,\ldots ,|Y_{bq}^{*}|\}$ denotes the maximum value of each of the random vectors $Y_{b}^{*}$ (in absolute value). Finally, the unknown quantile $z_{1-\alpha }(\max)$ is replaced with the observable estimator $y_{1-\alpha }^{*}(\max)$ in Equations (4) to (6), respectively. Note that the quantile $y_{1-\alpha }^{*}$ can also be computed directly using the *R*-function *qmvnorm* implemented in the *R*-package *mvtnorm*^20^ (if the dimension is not “too large”).

However, the present small sample sizes arise the question whether the method accurately controls the type-1 error rate and thus leads to reliable conclusions. Note that, in the data example, a 36 × 36 dimensional correlation matrix is estimated upon $n_{1}=10$ and $n_{2}=9$ independent vectors per group. Roughly speaking, the estimator might be too inaccurate when sample sizes are so small. In order to answer this question, a motivating simulation study has been conducted. Data has been generated from (i) multivariate normal and (ii) multivariate *T*_3_-distributions with *df *=* *3 degrees of freedom each with group specific covariance matrices ${\hat{V}}_{i}$, i.e.

(11) $$(i)\;X_{ik}∼N(0,{\hat{V}}_{i})\;\text{ and }\;(ii)\;X_{ik}∼T_{3}(0,{\hat{V}}_{i}),\;\;i=1,2;k=1,\ldots ,n_{i}$$

Here, ${\hat{V}}_{i}$ denotes the empirical covariance matrix of group *i* in the Alzheimer’s disease study. Data have been transformed to $Y_{ik}\prime =X_{ik}\prime \left({⊕}_{s=1}^{6}P_{6}\right)$ as described above. The *T*_3_-distribution is heavy tailed and might be a reasonable candidate to mimic the distributional shape of the protein abundance data. Note that ${\hat{V}}_{i}$ is singular and therefore data have been generated using singular value decomposition of ${\hat{V}}_{i}$ using the *rmvnorm* function implemented in the *mvtnorm* R-package.^21^ The simulation results for varying sample sizes $n_{1}=n_{2}=8,9,\ldots ,50$ at nominal significance level α=5% are displayed in Table 1.

**Table 1.** Type-1 error (α=5%) simulation results of the simulation-based test.^14^

Dist$\backslash n_{i}$	8	9	10	15	20	30	40	50
Nor	0.2134	**0.1908**	0.1723	0.1216	0.0975	0.0793	0.0720	0.0651
*T*_3_	0.1681	**0.1483**	0.1133	0.0873	0.0781	0.0647	0.0430	0.0420

significance is provided with alpha = 5%

It follows that the procedure does not control the type-1 error rate appropriately when sample sizes are very small. With sample sizes *n_i_* = 9, the empirical type-1 error rate is about 20% under normality and about 15% under heavy tailed $T_{3}(0,{\hat{V}}_{i})$-distribution and hence highly inflated. Only with larger sample sizes ($n_{i}\geq 50$), the method controls the type-1 error rate quite appropriately under normality, while it tends to be slightly conservative under $T_{3}(0,{\hat{V}}_{i})$, respectively. Digging for the reasons of this behavior, we first find that the procedure does not take the variations of the variance estimators used in the *t*-statistics in Equation (3) into account and second, the resampling algorithm is based on estimating the full correlation matrix of the vector of *t*-statistics. If a different resampling algorithm could be defined that overcomes both of these characteristics, a major improvement of the approximation might be available. In the next section, such a solution will be proposed.

## 4 Approximating the distribution of *T*_0_

The arising challenge is finding a *good* approximation of the joint distribution of T for estimating critical- and *p*-values. Resampling methods as above are an innovative way to do so. Roughly speaking, the corresponding test will work, if both the limiting and the resampling distributions of the statistic coincide—at least asymptotically—under the null hypothesis of no treatment effect. As explained above, the vector of *t*-test type statistics follows, asymptotically, a multivariate normal distribution with expectation 0 and correlation matrix R in low-dimensional settings (*d* fixed). This means that a proper resampling algorithm must be designed in such a way that the resampling distribution of T, say $T^{*}$, converges to the N(0,R) distribution, respectively, where the correlation matrix must be identical to the one defined in Equation (8). Moreover, in high-dimensional settings (with d→∞) similar observations apply, see the supplementary material, where it is, for example, shown that the distribution of T converges to a discrete Gaussian process. Detailed assumptions, especially on the covariance matrices, are listed in the supplementary material document as well. Having these thoughts in mind, not every resampling method is applicable in high-dimensional designs with an emphasis on small sample sizes. For example, the nonparametric bootstrap (drawing with replacement) shows poor finite sample performances in a similar setting under stronger conditions.^22^ Moreover, the therein proposed permutation method for exchangeable designs is in general not applicable in our unbalanced heteroscedastic setting.^23,24^ For more details on permutation tests, we refer to existing overviews and monographs.^25–28^ Therefore, the generation of the resampling variables and especially the algorithmic build-up play an important role for achieving a adequate bootstrap test in high-dimensional designs. Furthermore, even in low-dimensional settings (*d* fixed), estimating the approximate null N(0,R) distribution of T using a plug-in estimator $\hat{R}$ usually requires large sample sizes for an appropriate approximation. We therefore propose to approximate the limiting distribution of T without estimating the parameter of the distribution using a Wild-bootstrap randomization approach that is applicable in low- as well as high-dimensional situations. The method follows the same ideas proposed for matched pairs^18,29^ and in high-dimensional linear models,^30^ and is as follows. Let

$$Z_{ik}=Y_{ik}-{\bar{Y}}_{i\cdot },\;i=1,2;\;k=1,\ldots ,n_{i}$$

denote the centered random vectors and let *W_ik_* denote *N* independent and identically distributed random signs with $P(W_{ik}=\pm 1)=\frac{1}{2}$. Now, let

$$Z_{ik}^{*}=W_{ik}Z_{ik}$$

denote the resampling variables, ${\bar{Z}}_{i\cdot }^{*}=\frac{1}{n_{i}}\sum_{k=1}^{n_{i}}Z_{ik}^{*}=({\bar{Z}}_{i1\cdot }^{*},\ldots ,{\bar{Z}}_{iq\cdot }^{*})\prime$ their empirical means and let

(12) $${\hat{v}}_{i,\ell \ell }^{*}=N\frac{1}{n_{i}-1}\sum_{k=1}^{n_{i}}{\left(Z_{i\ell k}^{*}-{\bar{Z}}_{i\ell \cdot }^{*}\right)}^{2}\;,\;i=1,2\text{ and }\ell =1,\ldots ,q$$

denote the empirical variance of the variables obtained under the ℓth condition. Now, the resampling version of the original test statistic *T*_0_ is given by

(13) $$T_{0}^{*}=\max\{|T_{1}^{*}|,\ldots ,|T_{q}^{*}|\},\;\text{ where }\;T_{\ell }^{*}=\sqrt{N}\frac{\left({\bar{Z}}_{1\ell \cdot }^{*}-{\bar{Z}}_{2\ell \cdot }^{*}\right)}{\sqrt{{\hat{v}}_{1,\ell \ell }^{*}/n_{1}+{\hat{v}}_{2,\ell \ell }^{*}/n_{2}}}$$

In comparison to the existing methodology discussed in Section 3, the statistic $T_{0}^{*}$ mimics the computational process that lead to the original statistic *T*_0_. Moreover, it is shown in the supplementary material that for both low- and high-dimensional settings, the conditional distribution of the vector of statistics (given the data X)

(14) $$T^{*}=(T_{1}^{*},\ldots ,T_{q}^{*})\prime$$

mimics the null distribution of T. For making statistical inference, the critical value $z_{1-\alpha }(\max)$ is now estimated by the following steps:

1. Fix the data X (or Y) and compute the centered variables $Z_{ik}$.
2. Generate random weights *W_ik_*, compute the resampling variables $Z_{ik}^{*}$, the test statistics $T^{*}$ and safe the value of $T_{0}^{*}$ in $T_{0,b}^{*}$.
3. Repeat the previous step a large number of times (e.g. *M* = 10, 000) and compute the values $T_{0,1}^{*},\ldots ,T_{0,M}^{*}$.
4. Estimate $z_{1-\alpha }(\max)$ by the empirical (1−α)-quantile $z_{1-\alpha }^{*}(\max)$ of $T_{0,1}^{*},\ldots ,T_{0,M}^{*}$.

Finally, the unknown quantile $z_{1-\alpha }(\max)$ is replaced with the observable value $z_{1-\alpha }^{*}(\max)$ in Equations (4) to (6), respectively. One-sided tests and *p*-values are estimated analogously. The estimation of $z_{1-\alpha }(R)$ thus gets by without estimating the full correlation matrix R and additionally takes the variability of the variance estimators into account. Note that the set $\left\{H_{0}^{\left(\ell \right)},T_{\ell },\ell =1,\ldots ,q\right\}$ consisting of the null hypotheses and corresponding test statistics constitutes a joint testing family in the sense of Gabriel.^31^ Therefore, the simultaneous test procedure controls the family-wise error rate in the strong sense asymptotically in case of fixed *q*. Its accuracy in terms of controlling the type-1 error rate and power to detect alternatives when sample sizes are small will be investigated in the next section.

**Remark**: Throughout the manuscript, we consider the maximum statistic *T*_0_ as a combination of the *q* possibly correlated test statistics only. It originates from finding appropriate real valued $c_{1-\alpha }$ such that

$$P\left(\overset{q}{\underset{\ell =1}{\cap }}\left\{-c_{1-\alpha }\leq T_{\ell }\leq c_{1-\alpha }\right\}\right)=1-\alpha \Leftrightarrow P\left(\max_{\ell =1}^{q}|T_{\ell }|\leq c_{1-\alpha }\right)=1-\alpha$$

The right-hand side holds with $z_{1-\alpha }(\max)=c_{1-\alpha }$. The resulting test further allows inversion of the test statistics into simultaneous confidence intervals. However, we note that other combining functions than computing the maximum statistic would be possible, for instance Fisher’s weighted combining function. A general overview of nonparametric combination terminologies are provided in the monographs of Pesarin and Salmaso^32^ and Salmaso et al.^27^ and the references therein.

## 5 Simulations

In this section, we investigate the small sample properties of the proposed randomization technique within extensive simulation studies. The study aims to compare the two different approximations of the distribution of *T*_0_ presented in the paper. As the true distribution of *T*_0_ remains unknown, the type-1 error control of the competing methods will be used as a quality criterion. Later, the all-pairs and the any-pairs powers of the two methods will be compared. We conducted the extensive simulation studies in *R* (version 3.6.1). Marozzi^12^ discusses different methods to compute the numbers *nsim* and *nboot* of simulation and resampling runs in detail. Using his result and under some assumptions, *nsim *=* *10,000 simulations lead to $\mathrm{nboot}=8\sqrt{\mathrm{nsim}}=800$ resampling runs and a maximal simulation error of 0.006≈1%. Since the methods proposed in the manuscript are computationally feasible, we chose *nsim *=* *10,000 and *nboot *=* *1000 runs for each setting. Furthermore, setting α=5%, we can compute the 95% precision interval $\left[5\%\mp \frac{1.96}{\sqrt{1,000}}\sqrt{5\%*95\%}]=[4.6\%;5.4\%\right]$. If the empirical type-1 error of a test is within this interval, the method can be seen as accurate. The simulation code is displayed in the supplementary material file for reproducibility.

### 5.1 Type-1 error simulation results

Due to the abundance of possible factorial designs and hypotheses, two-way designs with varying dimension d∈{2,4,…,150} will be simulated and the hypothesis $H_{0}:P_{d}(\mu_{1}-\mu_{2})=0$ of *no interaction effect* will be tested at 5% level of significance. Data were generated from model

(15) $$X_{ik}∼F_{i}(\mu_{0},\Sigma_{i})+\mu_{i},\;i=1,2,\;k=1,\ldots ,n_{i},$$

where $F_{i}(\mu_{0},\Sigma_{i})$ represents a multivariate distribution with expectation vector $\mu_{0}$, correlation matrix $\Sigma_{i}$ and location shifts $\mu_{i}$. As representative marginal data distributions, we selected three differently tailed symmetric distributions (normal, logistic, *T*_3_) and three skewed distributions (ranging from mildly to very skewed) ($\chi_{7}^{2},\;\chi_{15}^{2}$, exponential) each with sample sizes $n_{i}\in \{10,20\}$. A major assessment criteria of the quality of the proposed approximations is the impact of both the chosen contrast as well as the dependency structures of the data—especially when data has different covariance matrices and thus covering a typical Behrens-Fisher situation. Here, we used normal copulas in order to generate rather complex dependency structures of the repeated measurements using the *R*-package *copula*.^33^ The different allocations of the correlation matrices used in the simulation studies are summarized in Table 2.

**Table 2.** Different correlation matrices used in the simulation study.

Setting 1:	$\Sigma_{1}=(\sigma_{1,ij})=0.6^{\left|i-j\right|}$	$\Sigma_{2}=(\sigma_{2,ij})=0.6^{\left|i-j\right|}$
Setting 2:	$\Sigma_{1}=(\sigma_{1,ij})=0.6^{\left|i-j|/(d-1\right)}$	$\Sigma_{2}=(\sigma_{2,ij})=0.6^{\left|i-j\right|}$
Setting 3:	$\Sigma_{1}=(\sigma_{1,ij})=1-|i-j|/d$	$\Sigma_{2}=(\sigma_{2,ij})=0.6^{\left|i-j|/(d-1\right)}$
Setting 4:	$\Sigma_{1}=I_{d}+0.5\cdot (J_{d}-I_{d})$	$\Sigma_{2}=I_{d}+0.25\cdot (J_{d}-I_{d})$.

In Setting 1, both correlation matrices $\Sigma_{1}$ and $\Sigma_{2}$ are identical and represent an autoregressive structure. In Settings 2 and 3, the covariance matrices $\Sigma_{1}$ and $\Sigma_{2}$ have different off-diagonal elements models, whereas an autoregressive structure depending on the dimension *d* is modeled by $\Sigma_{1}$ in Setting 2, and a linearly decreasing (symmetric) Toeplitz structure is covered by $\Sigma_{1}$ in Setting 3 (see Table 2), see Pauly et al.^7^ for similar choices. Note that Setting 2 models a pretty extreme scenario. For a detailed overview of copulas, we refer to Nelsen^34^ or Marozzi.^35^

**Figure 2.**
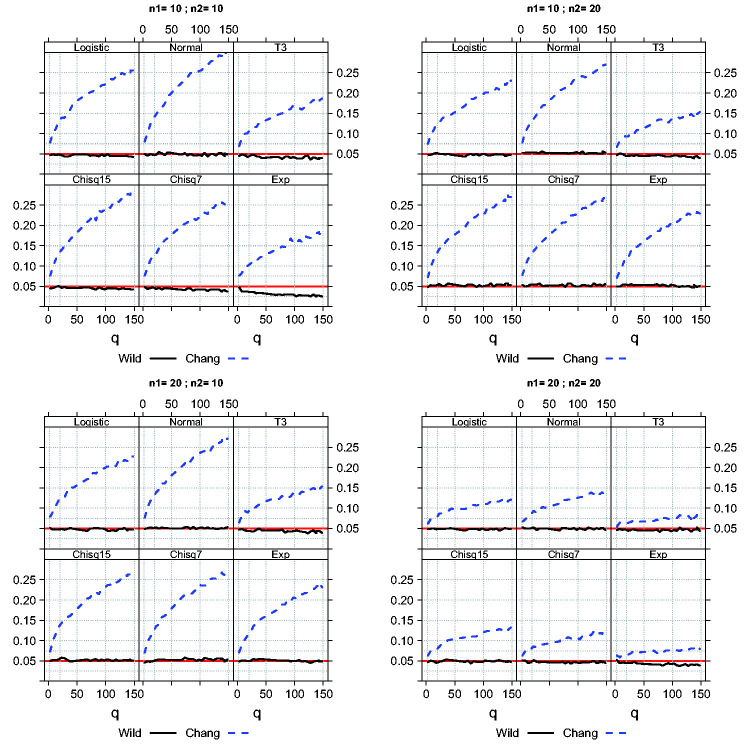
Type-1 error (α=5%) simulation results of the Wild-bootstrap randomization test T in Equation (14) (*Wild*) and simulation-based test T in Equation (10) (*Chang*). Data have covariance matrices as described in Setting 1 in Table 2.

All these four settings will be simulated for all four sample sizes ($n_{i}\in \{10,20\}$), dimensions (d∈{2,4,…,150}) and distributional configurations as described above. The type-1 error simulation results obtained under Setting 1 are displayed in Figure 2. All others are displayed in the supplementary material file.

First, it can be seen that the underlying covariance matrices significantly impact the accuracy of the simulation-based procedure proposed by Chang et al.^14^ in small sample size situations. It can also readily be seen that this test shows an increasing liberal behavior for increasing dimension *d*. The over-rejection of the hypotheses occurs, because the test decision is based upon quantiles from the $N(0,\hat{R})$ distribution, which neither takes the variability nor the distribution of the variance estimators into account. On the contrary, the randomization-based test *T*_0_ in Equation (13) tends to control the nominal type-1 error rate very well, even in case of very small sample sizes and large dimensions. The underlying covariance structures seem to impact the results only minor (if even). In case of mildly skewed distributions, the simulation results indicate that the resampling test controls the type-1 error accurately. However, in case of skewed data with different covariance matrices, the test might be very liberal when sample sizes are small. This behavior especially depends on the type of contrast of interest and whether it induces positive or negative correlations. The simulation results obtained under Setting 2 (see the supplementary material) indicate that none of the methods should be applied in these (rather extreme) cases in practice. Other methods, e.g. nonparametric methods rather than mean-based procedures, might be more appropriate for the analysis of small skewed data in such cases. However, the liberality vanishes with increasing sample sizes.^36^ In the three other settings considered here, the randomization procedure controls the type-1 error rate accurately, even when sample sizes are small and data follow skewed distributions. Pauly et al.^24^ report similar conclusions for general linear models with independent observations. Furthermore, in all of the settings considered here, the randomization-based resampling method is accurate when data is heavy-tailed but symmetric. As many factors might impact the behavior of the tests, however, the procedure might be sensitive to such distributional shapes in different scenarios than the ones considered here. For the generation of other copula models, e.g. Elliptical and Archimedean copulas, we refer to Marozzi.^35^

**Remark:** Instead of using copulas for generation of the multivariate distributions, an alternative method is generating data from model

$$X_{ik}=\mu_{i}+\Sigma_{i}^{-1/2}\epsilon_{ik},\;i=1,2,\;k=1,\ldots ,n_{i}$$

where the error terms is generated from standardized distributions, respectively. Additional s''imulation studies indicate that the empirical behavior of the test procedures is very similar.

### 5.2 Power simulation results

Next the *all-pairs* power *P* (“reject all false null hypotheses”) as well as the *any-pairs* powers *P* (“reject any true or false null hypothesis”) of the competing methods to reject the null hypothesis $H_{0}:P_{d}(\mu_{1}-\mu_{2})=0$ (α=5%) will be simulated for selected alternatives. The aim of the simulation study is investigating the impact of the underlying distributions, dependency structures of the data, sample size allocations and dimensions on the powers of the tests. Data have been generated (under the alternative) from model Equation (15) with expectations

$$\mu_{1}=(\delta ,2\delta ,\delta ,0,\ldots ,0)\prime \;\text{ and }\;\mu_{2}=(2\delta ,\delta ,2\delta ,0,\ldots ,0)\prime$$

and varying δ∈{0,0.1,…,2} from the same six distributions as above (normal, logistic, *T*_3_, $\chi_{7}^{2},\;\chi_{15}^{2}$ and exponential) having all of the four different dependency structures displayed in Table 2, respectively. The dimension of the random vectors was set to *d *=* *30. Due to the liberality of Chang et al.’s method for small sample sizes, large sample sizes were simulated (*n_i_* = 100) in order to be able to compare the powers of the methods on a fair basis, i.e. in a situation where both of them control the type-1 error rate accurately. For illustration, an additional power simulation with small sample sizes (*n_i_* = 10) has been conducted. First, it turns out that the types of covariance structures affect the powers of the tests. This is not surprising, because the higher the correlation the smaller are the variances of the effect size estimators. Overall, the simulation results indicate that the competing methods have comparable powers when sample sizes are large. Chang et al.’s method has slightly larger any-pairs and all-pairs powers than the randomization test (about 1% higher). However, when sample sizes are small, the randomization method controls the size and has a reasonable power. The simulations of the all-pairs power furthermore indicate the strong control of the FWER of both methods. Under the situations considered here, the shapes of the underlying distributions impact the results. As expected, the power of the methods under $\chi^{2}$-distributions appears to be rather low. The reason is the pretty large variance of the $\chi^{2}$-distribution compared with the other distributions. It should be noted that the above findings only hold for the settings considered here and might be different under other scenarios. The all-pairs and the any-pairs power curves are displayed in the supplementary material file.

## 6 Evaluation

The extensive simulation studies show that the newly proposed randomization test controls the type-1 error rate very satisfactorily, even when sample sizes are very small and data do not follow a multivariate normal distribution. In a first step, we perform a further type-1 error simulation study and investigate the accuracy of the method for analyzing this specific data set in the same way as presented in Table 1. As before in Equation (11), we mimic the data set using multivariate normal $N(0,{\hat{V}}_{i})$ and multivariate $T_{3}(0,{\hat{V}}_{i})$ distributions, respectively. The type-1 error simulation results are displayed in Table 3. It appears that the method controls the type-1 error accurately. The high-dimensional preclinical study on Alzheimer’s disease introduced in Section 2 can therefore now be analyzed with this method. For comparisons, the data set will be analyzed using both of the discussed approximations.

**Table 3.** Type-1 error (α=5%) simulation results of the Wild-bootstrap randomization test.

Dist$\backslash n_{i}$	8	9	10	15	20	30	40	50
Nor	0.0530	**0.0512**	0.0494	0.0496	0.0474	0.0502	0.0501	0.0497
*T*_3_	0.0392	**0.0411**	0.0431	0.0464	0.0458	0.0512	0.0527	0.0502

significance is provided with alpha = 5%

In addition to testing for interactions motivated in Equation (2), multiple comparisons inferring the region as well as the group effects are of interest. These will be performed using the contrast matrices

$$H_{0}^{\mu }:C\mu =0,\;\text{ where }\;C=\left({⊕}_{s=1}^{6}P_{6}\right)\left(I_{36}\vdots \;I_{36}\right)\;\;\text{(Region)},\;\;\;\text{ and }$$

(16) $$H_{0}^{\mu }:C\mu =0,\;\text{ where }\;C=\left(I_{36}\vdots -I_{36}\right)\;\;(\text{Group})$$

Note that for testing the impact of the region, the test statistics are given by

$$T_{\ell }=\sqrt{N}\frac{{\bar{Y}}_{1\ell \cdot }+{\bar{Y}}_{2\ell \cdot }-(\theta_{1\ell }+\theta_{2\ell })}{\sqrt{{\hat{v}}_{1,\ell \ell }/n_{1}+{\hat{v}}_{2,\ell \ell }/n_{2}}}$$

where ${\bar{Y}}_{i\ell \cdot }$ denotes the mean of the ℓth component of the vector $Y_{ik}\prime =X_{ik}\prime \left({⊕}_{s=1}^{6}P_{6}\right)$. Therefore, the correlation matrix of the vector of test statistics $T=(T_{1},\ldots ,T_{q})\prime$ is identical to the one using interaction contrasts as described in Section 3. The randomization approach for approximating the joint null distribution of these statistics is adapted accordingly. Testing for the group effects is the “standard” multivariate hypothesis. Means and empirical variances of the protein abundance data under each protein × region × group combination are provided in the supplementary material file.

As already indicated by the confidence interval plots in Figure 1, data show a fairly amount of variance heteroscedasticity. Therefore, assuming equal covariance matrices across the groups is doubtful. Next, the multiple hypotheses will be tested using the two different approaches. The point estimators of the contrasts in means ${\hat{\delta }}_{\ell }$, values of the test statistics $T_{\ell }$ equation (3), the estimated quantile $z_{95\%}(\max)$ as well as 95%-simultaneous confidence intervals equation (5) using both the simulation as well as randomization technique will be displayed for all of the three multiple hypotheses. In total, *M *=* *100,000 simulation and randomization runs have been performed. The results are displayed in Table 4. Different decisions (at 5% level) between the two competing methods are highlighted in boldface.

**Table 4.** Multiple contrast test results of the protein abundance trial.

	Interaction						Region						Group					
	Chang ($z_{95\%}=3.10)$			Randomization ($z_{95\%}=3.76$)			Chang ($z_{95\%}=3.10)$			Rand. ($z_{95\%}=3.75$)			Chang ($z_{95\%}=3.09)$			Rand. $\left(z_{95\%}=3.88\right)$		
ℓ=	${\hat{\delta }}_{\ell }$	$T_{\ell }$	95%-L	95%-U	95%-L	95%-U	${\hat{\delta }}_{\ell }$	$T_{\ell }$	95%-L	95%-U	95%-L	95%-U	${\hat{\delta }}_{\ell }$	$T_{\ell }$	95%-L	95%-U	95%-L	95%-U
1	−0.65	−1.20	−2.32	1.03	−2.67	1.37	1.35	2.50	−0.32	3.03	−0.68	3.38	0.39	0.41	−2.57	3.36	−3.32	4.11
2	−0.53	−1.00	−2.17	1.11	−2.50	1.44	2.49	4.71	0.85	4.13	0.51	4.48	0.51	0.69	−1.77	2.80	−2.35	3.38
3	1.09	1.37	−1.37	3.55	−1.88	4.05	−2.38	−3.00	−4.84	0.07	−5.36	0.59	2.13	1.93	−1.28	5.55	−2.15	6.42
4	−0.04	−0.10	−1.41	1.32	−1.69	1.60	−2.40	−5.43	−3.77	−1.03	−4.06	−0.74	1.00	1.06	−1.92	3.91	−2.66	4.66
5	−0.01	−0.02	−1.39	1.37	−1.67	1.66	−0.68	−1.53	−2.06	0.70	−2.36	0.99	1.04	1.03	−2.08	4.15	−2.87	4.94
6	0.14	0.28	−1.41	1.70	−1.74	2.02	1.62	3.23	**0.06**	**3.18**	−**0.26**	**3.51**	1.18	1.20	−1.85	4.21	−2.62	4.99
7	−0.22	−0.72	−1.15	0.71	−1.34	0.91	0.18	0.58	−0.76	1.11	−0.95	1.30	−0.61	−1.84	−1.64	0.41	−1.90	0.68
8	−0.68	−2.50	−1.53	0.16	−1.71	0.34	−1.85	−6.75	−2.70	−1.00	−2.88	−0.82	−1.08	−2.90	−2.23	0.07	−2.53	0.36
9	0.15	0.74	−0.49	0.80	−0.63	0.94	−0.10	−0.50	−0.75	0.54	−0.89	0.68	−0.24	−0.89	−1.09	0.60	−1.30	0.82
10	0.20	0.98	−0.43	0.83	−0.56	0.96	−1.97	−9.69	−2.60	−1.34	−2.74	−1.21	−0.20	−0.85	−0.91	0.52	−1.10	0.70
11	0.24	1.41	−0.28	0.75	−0.39	0.86	1.10	6.58	0.58	1.62	0.47	1.73	−0.16	−0.61	−0.98	0.66	−1.19	0.87
12	0.31	2.42	−0.09	0.71	−0.17	0.79	2.65	20.58	2.25	3.05	2.17	3.13	−0.09	−0.77	−0.43	0.25	−0.51	0.34
13	0.46	1.44	−0.53	1.45	−0.74	1.66	2.62	8.18	1.63	3.62	1.42	3.83	0.59	1.66	−0.51	1.69	−0.79	1.97
14	−0.07	−0.31	−0.79	0.64	−0.93	0.79	3.73	16.17	3.02	4.45	2.87	4.60	0.06	0.24	−0.70	0.82	−0.90	1.02
15	−0.23	−0.29	−2.67	2.21	−3.18	2.72	−6.67	−8.45	−9.12	−4.23	−9.63	−3.71	−0.10	−0.09	−3.37	3.17	−4.20	4.00
16	−0.41	−1.80	−1.13	0.30	−1.27	0.45	−3.98	−17.27	−4.69	−3.26	−4.84	−3.11	−0.28	−0.62	−1.70	1.14	−2.07	1.50
17	0.24	0.55	−1.11	1.59	−1.39	1.87	1.46	3.34	**0.11**	**2.81**	−**0.18**	**3.10**	0.37	0.98	−0.80	1.54	−1.10	1.84
18	0.01	0.04	−0.91	0.94	−1.10	1.13	2.83	9.47	1.90	3.75	1.71	3.95	0.14	0.45	−0.84	1.13	−1.09	1.38
19	−0.57	−1.11	−2.14	1.01	−2.47	1.34	−1.78	−3.49	−3.36	−0.20	−3.69	0.13	−0.86	−1.41	−2.74	1.02	−3.22	1.49
20	−1.08	−1.89	−2.86	0.69	−3.22	1.05	−0.50	−0.88	−2.28	1.27	−2.65	1.64	−1.38	−1.83	−3.70	0.94	−4.29	1.53
21	0.54	1.62	−0.49	1.56	−0.70	1.77	0.58	1.75	−0.44	1.61	−0.66	1.82	0.24	0.76	−0.74	1.23	−0.99	1.48
22	−0.17	−0.56	−1.14	0.79	−1.34	0.99	−3.14	−10.09	−4.10	−2.17	−4.30	−1.97	−0.47	−1.52	−1.42	0.48	−1.66	0.72
23	0.72	1.35	−0.93	2.37	−1.27	2.71	1.05	1.97	−0.60	2.70	−0.95	3.05	0.43	0.62	−1.70	2.55	−2.24	3.09
24	0.57	2.32	−0.19	1.32	−0.35	1.48	3.79	15.52	3.03	4.55	2.87	4.71	0.27	0.86	−0.70	1.25	−0.95	1.49
25	0.45	1.16	−0.75	1.65	−1.00	1.90	3.31	8.52	2.11	4.52	1.85	4.77	−0.16	−2.19	−0.39	0.07	−0.45	0.13
26	0.30	0.72	−1.00	1.61	−1.27	1.88	2.80	6.63	1.49	4.11	1.21	4.38	−0.31	−2.55	−0.69	0.07	−0.78	0.16
27	−0.92	−1.93	−2.39	0.56	−2.70	0.86	−2.17	−4.56	−3.65	−0.70	−3.96	−0.38	−1.53	−2.05	−3.84	0.78	−4.43	1.37
28	−0.76	−1.40	−2.44	0.92	−2.78	1.26	−5.50	−10.16	−7.18	−3.83	−7.54	−3.47	−1.37	−1.54	−4.13	1.38	−4.83	2.08
29	0.59	2.64	−0.10	1.28	−0.25	1.43	2.05	9.18	1.36	2.75	1.21	2.89	−0.02	−0.08	−0.95	0.90	−1.18	1.13
30	0.33	0.86	−0.87	1.53	−1.11	1.78	−0.49	−1.26	−1.68	0.71	−1.94	0.96	−0.28	−0.39	−2.50	1.93	−3.07	2.50
31	0.11	0.52	−0.55	0.78	−0.69	0.92	0.45	2.08	−0.22	1.11	−0.36	1.26	0.40	1.87	−0.26	1.06	−0.43	1.23
32	−0.22	−0.93	−0.94	0.50	−1.09	0.65	1.07	4.59	0.35	1.79	0.20	1.94	0.07	0.37	−0.51	0.65	−0.66	0.80
33	0.31	0.96	−0.69	1.31	−0.89	1.52	−2.18	−6.77	−3.18	−1.18	−3.40	−0.97	0.60	1.52	−0.62	1.82	−0.93	2.12
34	0.22	0.81	−0.61	1.05	−0.79	1.22	−3.10	−11.54	−3.93	−2.27	−4.11	−2.09	0.51	1.60	−0.47	1.48	−0.72	1.73
35	−0.43	−1.81	−1.16	0.31	−1.32	0.46	0.85	3.59	**0.12**	**1.59**	−**0.04**	**1.75**	−0.14	−0.50	−1.00	0.72	−1.22	0.94
36	0.01	0.06	−0.41	0.42	−0.49	0.51	2.91	21.80	2.50	3.33	2.41	3.41	0.30	2.02	−0.16	0.75	−0.27	0.86
	$T_{0}=2.63$						$T_{0}=21.80$						$T_{0}=2.90$					

Here, ℓ=1,…,36 corresponds to each of the 36 contrasts tested by the hypothesis of no interaction, region or group as given in Equation (2) or Equation (16), respectively. In addition, ${\hat{\delta }}_{\ell }$ is the estimated contrast, $T_{\ell }$ the value of the *t*-test type statistic and 95%-L or 95%-U the 95% lower or upper bound of the 95% simultaneous confidence intervals. The estimated quantiles $z_{95\%}(\max)$ are given in the headers. Different test decisions between the competing methods are marked boldface.

First, for all of the three different testing problems, the estimated quantiles of the maximum statistic $z_{95\%}(\max)$ are way larger using the randomization approach than with the simulation-based method. This is not surprising when reflecting the liberal behavior of the test. The simultaneous confidence intervals are therefore wider using the randomization procedure. In the following, results obtained for each of the three multiple null hypotheses will be discussed separately. Neither of the two competing methods detects an interaction between group and region under any of the six investigated proteins. The estimated quantiles differ remarkably, though (3.10 vs. 3.76). But, since the maximum *t*-statistic is $T_{0}=2.63$ and thus $T_{0}<z_{95\%}(\max)$, data do not provide the evidence to reject the null hypothesis at 5% level of significance. Investigating differences in the regions, the approximation methods provide different local conclusions at 5% level of significance. The simulation-based method declares the regions ACB under Syntaxin, M1 under VAMP2 as well as M1 under Alpha-synuclein significantly different from the average of the others, while the randomization method does not. Taking a look at the boxplots give the impression that these values differ only slightly from the mean of the others. Clearly, given the amount of regional deviations, those regions differ significantly on a pairwise level. Also, overall, the protein abundances differ significantly across the regions. Investigating differences between the groups, no significant differences can be detected using any of the competing methods. It should be noted that the estimated quantile using the randomization method increases from 3.75 to a value of 3.88, while the simulation-based estimator is still about 3.10 (3.09).

## 7 Discussion and outlook

Research experiments in translational medicine and especially in preclinical areas are usually small due to ethical reasons and animal welfare. Clearly, animal studies should be abandoned, but, roughly speaking, medical research has not arrived at the point yet to replace and refine every experiment to avoid animals. Often, sample sizes of such trials are smaller than 20 per experimental group, which might be a reason to argue the quality of the outcome. However, since animals are kept under homogeneous conditions, heteroscedasticity across the animals is usually smaller compared to other scenarios in humans, depending on the outcome measures. Anyway, since preclinical studies play a significant role in medical sciences in terms of transferring the results towards the next phase, a major concern is the quality of the used statistical methods. Most of them control the type-1 error rate accurately with large sample sizes only and, indeed, they show a very liberal or conservative behavior when sample sizes are small. This observation holds for a variety of statistical procedures designed for different questions and fields, including analysis of variance methods^24,37^ as well as multiple contrast test procedures using maximum *t*-test type statistics^38,39^ for repeated measures and multivariate data. When the number of comparisons is “small” compared to the sample sizes, approximate and exact methods are available.^19,40–43^ These methods are, however, limited to the number of comparisons to be made and are not applicable in high-dimensional situations. Note that the methods are not applicable because of the test statistic itself (maximum *t*-test) or because of any computational difficulty, and information about its distribution is only available for low-dimensional designs. Recently, Chang et al.^14^ tackled the problem and proposed a simulation-based algorithm to approximate the distribution of the maximum statistic in high-dimensional designs. Extensive simulations show, however, that large sample sizes are needed for an accurate type-1 error rate control making their method not applicable for trials with small sample sizes. In the present paper, we modified their strategy towards a robust randomization technique to approximate the null distribution of the max *t*-test. Simulation studies indicate that the method approximates the null distribution very satisfactorily and greatly improves the applicability of their method.

Furthermore, the power of the method can be considerably improved by adapting it to a two-step screening procedure. For a given significance level *α*, define the index set

(17) $$S_{1}=\{1\leq \ell \leq q:|T_{\ell }|\leq \sqrt{2\log(q)}+{\left\{2\log(q)\right\}}^{-1/2}+\sqrt{2\log(1/(1-\alpha ))}\}$$

which contains the indices of all test statistics $T_{\ell }$ that—in absolute value—do not exceed the given bound in $S_{1}$. If the cardinality of the set is equal to *q*, the null hypothesis $H_{0}:\theta_{1}=\theta_{2}$ is not rejected. Otherwise, if its cardinality is equal to *s* (say) and smaller than *q* (*s *<* q*), select all corresponding test statistics in the sub-vector T˜={Ts,s∈S1}. The null hypothesis will be rejected, if max⁡{|Ts|,s∈S1}≥y1−α*(S1). Here, $y_{1-\alpha }^{*}(S_{1})$ denotes the (1−α)-quantile of the values max⁡s∈S1|Y1s*|,…,max⁡s∈S1|YMs*|, where the $Y^{*}$’s are defined in equation (10). Thus, the dimension of the testing problem is basically reduced to testing the null hypotheses $H_{0}^{\left(s\right)}:{\tilde{\theta }}_{1}={\tilde{\theta }}_{2}$, where the hypothesis matrix $\tilde{C}$ has appropriate dimensions and $\tilde{\mu }$ collects all corresponding *μ_i_*’s being excluded from $S_{1}$. Moreover, the dimension reduction implies that the critical value of the screening modification does not exceed the original one, i.e. $y_{1-\alpha }^{*}(S_{1})\leq y_{1-\alpha }^{*}$. As the value of the test statistic does not change (max⁡{|Ts|,s∈S1}=T0 by definition), screening indeed improves the power. For the same reason, however, screening might result in even more liberal test decisions than the original version without screening. In addition, the interpretation of the screening results may be challenging in case of arbitrary contrasts or especially interaction effects. For these two reasons, we did not consider an additional screening stage. Detailed power investigations and dimension reductions will be part of future research. All of the methods considered in the paper are based on means of data. Nonparametric methods, for instance based on ranks of the data, or simultaneous methods based on interpoint distances as well as investigating other combination functions than maximum,^12^ will be part of further investigations as well.

## Supplemental Material

sj-pdf-1-smm-10.1177_0962280220970228 - Supplemental material for Small sample sizes: A big data problem in high-dimensional data analysis Click here for additional data file. Supplemental material, sj-pdf-1-smm-10.1177_0962280220970228 for Small sample sizes: A big data problem in high-dimensional data analysis by Frank Konietschke, Karima Schwab and Markus Pauly in Statistical Methods in Medical Research
