# Supplementary material for: Small sample sizes: A big data problem in high-dimensional data analysis
Source: Stat Methods Med Res. 2020 Nov 24;30(3):687–701. doi: 10.1177/0962280220970228 (PMC8008424; doi:10.1177/0962280220970228)
Supplement: sj-pdf-1-smm-10.1177_0962280220970228 - Supplemental material for Small sample sizes: A big data problem in high-dimensional data analysis [file sj-pdf-1-smm-10.1177_0962280220970228.pdf]

Journal Title
XX(X):1–24

©The Author(s) 2020
Reprints and permission:
sagepub.co.uk/journalsPermissions.nav

DOI: 10.1177/ToBeAssigned
www.sagepub.com/
SAGE

Supplementary Material to: Small sample sizes: A big data problem in high-dimensional data analysis

Frank Konietschke[1] and Karima Schwab [2] and Markus Pauly [3]

1 More Details on the Set-Up

As explained in the main paper, our main achievement is the proposal of a resampling based multiple comparison procedure that is designed in a way that it is applicable in low- and high-dimensional settings while estimating the correlation matrix of the different test statistics is not necessary. We hereby distinguish the two different underlying asymptotic frameworks corresponding to low-and high-dimensional cases, both being motivated from statistical practice:

1. The number of experimental conditions (time points) d is fixed and the sample size $N \to \infty$.
2. The number of experimental conditions (time points) d may depend on N and is not a model constant, i.e. $d = d(N) \to \infty$ as $N \to \infty$.

[1]Charité- Universitätsmedizin Berlin, Corporate Member of Freie Universität Berlin, Humboldt-Universität zu Berlin, and Berlin Institute of Health, Institute of Biometry and Clinical Epidemiology, Charitéplatz 1, 10117 Berlin, Germany Berlin Institute of Health (BIH), Anna-Louisa-Karsch-Straße 2, 10178 Berlin, Germany
[2]Charité- Universitätsmedizin Berlin, Institute of Pharmacology, Charitéplatz 1, 10117 Berlin
[3]Technical University of Dortmund, Faculty of Statistics, Germany

Corresponding author:
Frank Konietschke
Email: Frank.Konietschke@charite.de

Note that the numbers of hypotheses to be tested may depend on d (e.g. in many-to-one comparisons) and thus may also diverge as indicated in Case 2. In this context an astonishing feature of our proposed resampling technique is that it allows for a simultaneous treatment of both cases.

Multiple contrast tests for the classical low-dimensional case with fixed d have been studied in detail under normality assumption, see, e.g.,[1] for $d \in \{3,4,5\}$ and[2–4] for general d. Procedures for other parametric models have been considered by[5] and implemented in the **R**-package multcomp[6]. Global testing procedures for high-dimensional repeated measures have been proposed by[7] and[8] for the one and two sample case and recently been extended by[?] for an arbitrary number of groups.

After this short prequel recall that we consider a multivariate two-sample design given by independent and identically distributed d-dimensional random vectors in $i = 1, 2$ independent groups

$$\boldsymbol{X}_{ik} = (X_{i1k}, \ldots, X_{idk})' \sim \boldsymbol{F}_i, \quad i = 1,2;\ k = 1, \ldots, n_i;\ N = n_1 + n_2, \tag{1.1}$$

with expectation $E(\boldsymbol{X}_{i1}) = \boldsymbol{\mu}_i = (\mu_{i1}, \ldots, \mu_{id})'$ and covariance matrix $Cov(\boldsymbol{X}_{i1}) = \boldsymbol{\Sigma}_i > 0, \quad i = 1, 2$. Set $\boldsymbol{\mu} = (\boldsymbol{\mu}_1', \boldsymbol{\mu}_2')'$. In this general model, Hypotheses of interest $H_0^\mu : \boldsymbol{C\mu} = \boldsymbol{0}$ are manifold, but can be described in a general way using a $q \times (2 \cdot d)$ dimensional linear contrast matrix

$$\boldsymbol{C} = \begin{pmatrix} \boldsymbol{c}_1' \\ \vdots \\ \boldsymbol{c}_q' \end{pmatrix} = \begin{pmatrix} c_{11} & \cdots & c_{1d} & c_{1(d+1)} & \cdots & c_{1(2d)} \\ c_{21} & \cdots & c_{1d} & c_{2(d+1)} & \cdots & c_{2(2d)} \\ \vdots & \ddots & \vdots & \vdots & \vdots & \vdots \\ c_{q1} & \cdots & \cdots & \cdots & \cdots & c_{q(2d)} \end{pmatrix}.$$

Each row of $\boldsymbol{C}$ represents one contrast and thus one comparison. Note that the global null hypothesis $H_0^\mu : \boldsymbol{C\mu} = \boldsymbol{0}$ can be equivalently written as

$$H_0^\mu : \boldsymbol{C\mu} = \boldsymbol{0} \Leftrightarrow \begin{cases} H_0^{(1)} : \boldsymbol{c}_1'\boldsymbol{\mu} = 0 \\ H_0^{(2)} : \boldsymbol{c}_2'\boldsymbol{\mu} = 0 \\ \qquad \vdots \\ H_0^{(q)} : \boldsymbol{c}_q'\boldsymbol{\mu} = 0 \end{cases} \Leftrightarrow \bigcap_{\ell=1}^{q} \left\{ H_0^{(\ell)} : \boldsymbol{c}_\ell'\boldsymbol{\mu} = 0 \right\}$$

and therefore, testing the global null $H_0^\mu : \boldsymbol{C\mu} = \boldsymbol{0}$ is basically equivalent to testing the local null hypotheses $H_0^{(\ell)}, \ell = 1, \ldots, q$, simultaneously at multiple level α. Which linear contrast matrix to use, however, depends on specific study questions and cannot be recommended in general. A few prominent examples are listed below:

(a) Multiple comparisons of the multivariate hypotheses $H_0^\mu : \boldsymbol{\mu}_1 = \boldsymbol{\mu}_2$ can be performed using $\boldsymbol{C} = (\boldsymbol{I}_d \vdots -1 \cdot \boldsymbol{I}_d)$.[9] investigate multiple comparisons for testing the aforementioned hypothesis in detail.

(b) Testing multiple interaction effects between the factors group and time can be

performed using $\boldsymbol{C} = \boldsymbol{P}_d(\boldsymbol{I}_d \vdots - 1\boldsymbol{I}_d)$. Here, $\boldsymbol{P}_d = (\boldsymbol{I}_d - \frac{1}{d}\boldsymbol{J}_d)$ denotes the so-called centering matrix.

(c) Main time effects can be tested using $\boldsymbol{C} = \boldsymbol{P}_d(\boldsymbol{I}_d \vdots \boldsymbol{I}_d)$.
(d) Many-to-one comparisons of the first component to all others per group can be conducted by $\boldsymbol{C} = (\mathbf{1}_{d-1} \vdots - \boldsymbol{I}_{d-1}) \oplus (\mathbf{1}_{d-1} \vdots - \boldsymbol{I}_{d-1})$.

2 Theoretical properties

In this section the theoretical properties of the introduced test statistics will be discussed. For the ease of notation, we concentrate on the multivariate testing problem $H_0 : \boldsymbol{\theta}_1 \pm \boldsymbol{\theta}_2 = \mathbf{0}$, where $\boldsymbol{\theta}_i = E(\boldsymbol{Y}_{ik})$ and $\boldsymbol{Y}_{ik} = \boldsymbol{C}\boldsymbol{X}_{ik}$, $i = 1, 2; k = 1, \ldots, n_i$. Since the numbers of contrasts q to be tested may depend on the dimension d, e.g. in Dunnett-type many-to-one comparisons, they play an important role in the asymptotic frameworks. We therefore distinguish between fixed and diverging numbers of comparisons:

- **Case A. (Low-Dimensional MCTP)** $\boldsymbol{C} = \boldsymbol{C}_q \in \mathbb{R}^{q \times d}$ in case of a fixed number $q < \infty$,
- **Case B. (High-Dimensional MCTP)** $\boldsymbol{C} = \boldsymbol{C}_r \in \mathbb{R}^{r \times d}$, where $r = r(d)$ and $d \to \infty$.

In the sequel we study the two different asymptotic frameworks, namely assuming that the sample size N goes to infinity, i.e. $N \to \infty$, and $d = d(N)$ is a subsequence thereof. This includes both the cases **A** and **B** of low- and high-dimensional designs, namely $d(N) \equiv d \in \mathbb{N}$ fixed as well as $d = d(N) \to \infty$ simultaneously with $N \to \infty$. In the latter, we do not impose any specific relation between N and d, i.e. the high-dimensional setting with $d > N$ is automatically included. For the existence of Gaussian limit distributions the following regularity conditions on the covariance matrices are imposed (corresponding to the choice of $\boldsymbol{C}$):

Case A Let $N \to \infty$, $d \to \infty$ and let the numbers of comparisons q in $\boldsymbol{C} = \boldsymbol{C}_q \in \mathbb{R}^{q \times d}$ be fixed such that

$$\boldsymbol{C}\boldsymbol{\Sigma}_i\boldsymbol{C}' \;\to\; \boldsymbol{V}_i \in \mathbb{R}^{q \times q}, \;\; \text{with } \boldsymbol{V}_i = \boldsymbol{V}_i' > 0, \; i = 1, 2.$$

Case B Let $N \to \infty$, $d \to \infty$ and let the numbers of comparisons $q = q(d)$ in $\boldsymbol{C} = \boldsymbol{C}_q \in \mathbb{R}^{q \times d}$ grow with increasing dimension d such that

$$\boldsymbol{C}\boldsymbol{\Sigma}_i\boldsymbol{C}' \;\to\; \boldsymbol{V}_i \in \mathbb{R}^{\mathbb{N} \times \mathbb{N}}, \;\; \text{with } \boldsymbol{V}_i = \boldsymbol{V}_i' > 0, \; i = 1, 2 \text{ and}$$

$$\sup_{i,\ell}(v_{i,\ell\ell}) < \infty, \;\; \text{where } v_{i,\ell\ell} \text{ denotes the } \ell\text{-th diagonal element of } \boldsymbol{V}_i.$$

The additional condition in **Case B** is needed to guarantee that all variances are bounded. In most practical applications in the life sciences this is not a restrictive condition. Moreover, in both of the **Cases A** and **B**, we assume that

$$\frac{N}{n_i} \to \lambda_i < \infty, \; i = 1, 2; \; N \to \infty, \tag{2.2}$$

Let $\overline{\boldsymbol{Y}}_{i\cdot}$ denote the vector of means in group i and note that $Cov(\boldsymbol{Y}_{ik}) = \boldsymbol{C}\boldsymbol{\Sigma}_i\boldsymbol{C}'$ and $Cov(\sqrt{N}\,\overline{\boldsymbol{Y}}_{i\cdot}) = \frac{N}{n_i}\boldsymbol{C}\boldsymbol{\Sigma}_i\boldsymbol{C}'$. Using this notation we define the statistic

$$\boldsymbol{U}_N \;=\; \sqrt{N}(\overline{\boldsymbol{Y}}_{1\cdot} \pm \overline{\boldsymbol{Y}}_{2\cdot}), \tag{2.3}$$

the covariance matrix of which is given by

$$\boldsymbol{S}_N \;=\; Cov(\boldsymbol{U}_N) \;= \sum_{i=1}^{2} \frac{N}{n_i}\boldsymbol{C}\boldsymbol{\Sigma}_i\boldsymbol{C}'. \tag{2.4}$$

Next we establish the asymptotic distribution of the statistics $\boldsymbol{U}_N$ in both asymptotic frameworks, i.e. assuming fixed and increasing numbers of contrasts.

Theorem 1. *Under the respective assumptions of **Cases A**, **B** and under the Assumption* (2.2)*,*

$$\boldsymbol{U}_N - \sqrt{N}(\boldsymbol{\theta}_1 \pm \boldsymbol{\theta}_1) \xrightarrow{d} \begin{cases} N_q(\boldsymbol{0}_q, \boldsymbol{V}_q), & \textbf{\textit{Case A}}, \\ N_\infty(\boldsymbol{0}_\infty, \boldsymbol{V}_\infty), & \textbf{\textit{Case B}}, \end{cases} \tag{2.5}$$

where $\boldsymbol{V}_q = \sum_{i=1}^{2} \lambda_i \boldsymbol{V}_i$ and $\boldsymbol{V}_\infty = \sum_{i=1}^{2} \lambda_i \boldsymbol{V}_i$ are limiting covariance matrices.

REMARK 2.1. *Note that the result in **Case B** means that we have convergence in distribution on $\mathbb{R}^{\mathbb{N}}$ of the random process $((\boldsymbol{U}_N - \sqrt{N}(\boldsymbol{\theta}_1 \pm \boldsymbol{\theta}_1))', \boldsymbol{0}'_\infty)'$ to a zero-mean Gaussian-process with covariance function $\xi(k, \ell) = v_{k\ell}, k, \ell \in \mathbb{N}$ where $v_{k\ell}$ is the (k, ℓ)-th entry of $\boldsymbol{V}_\infty$.*

However, as discussed above, raw means are usually not the preferred choice of a test statistic in practice rather than studentized means. The latter are unit free and therefore do not depend on data scales. Here, each component of the statistic $\boldsymbol{U}_N$ is studentized with its corresponding variance estimator. In order to derive the asymptotic joint distribution of the vector of test statistics $\boldsymbol{T}$, the following Lemma is needed.

Lemma 1. *Under the Assumption* (2.2)

$$\sup_{i,\ell} |\widehat{v}_{i,\ell\ell} - v_{i,\ell\ell}| \xrightarrow{p} 0 \;\; \textit{in probability as } N \to \infty.$$

Next, the asymptotic distribution of $\boldsymbol{T}$ will be established in the next theorem.

Theorem 2. *Under the respective assumptions of **Cases A**, **B** and under the Assumption* (2.2)*,*

$$\boldsymbol{T} \xrightarrow{d} \begin{cases} N_q(\boldsymbol{0}_q, \boldsymbol{R}_q), & \textbf{\textit{Case A}}, \\ N_\infty(\boldsymbol{0}_\infty, \boldsymbol{R}_\infty), & \textbf{\textit{Case B}}, \end{cases} \tag{2.6}$$

where $\boldsymbol{R}_\infty = (v_{k\ell}/\sqrt{v_{kk}v_{\ell\ell}})_{k,\ell}, k, \ell \in \mathbb{N}$, denotes the asymptotic correlation function.

It follows from both of the two Theorems 1 and 2 that the limiting distributions of the means and the t-test type statistics follow multivariate normal distributions (**Case A**) or Gaussian processes (**Case B**), respectively.

In the next Theorem we show that the conditional distribution of $\boldsymbol{T}^*$ coincides with the asymptotic null-distribution of $\boldsymbol{T}$.

Theorem 3. *Given the data $\boldsymbol{X}_{ik}$, $i = 1, 2$; $k = 1, \ldots, n_i$, it holds under the the Assumption (2.2) that for all $\boldsymbol{\mu} \in \mathbb{R}^{2d}$ the resampling statistic $\boldsymbol{T}^*$ has, asymptotically ($N \to \infty$) in probability, the same distribution as $\boldsymbol{T}$ in Theorem 2 under the null hypothesis $H_0 : \boldsymbol{\theta}_1 \pm \boldsymbol{\theta}_2 = \mathbf{0}$ in both **Case A** and **Case B**.*

REMARK 2.2.
(a) This result implies that the proposed randomization approach is consistent in the sense of approximating the null distribution of the test statistic $\boldsymbol{T}$.
(b) Let ρ_q and ρ_∞ denote a distance, e.g. the Prohorov distance (see Dudley, 2001), that metrizes weak convergence on $\mathbb{R}^q$ and $\mathbb{R}^{\mathbb{N}}$, respectively. Moreover, denote by $\mathcal{L}(\boldsymbol{T}|H_0)$ the distribution of $\boldsymbol{T}$ under the null hypothesis and $\mathcal{L}(\boldsymbol{T}^*|\mathbf{X})$ the conditional distribution of $\boldsymbol{T}^*$ given the data. Then Theorem 3 states that

$$\rho_q(\mathcal{L}(\boldsymbol{T}^*|\mathbf{X}), \mathcal{L}(\boldsymbol{T}|H_0)) \stackrel{p}{\longrightarrow} 0 \quad \textit{in } \textbf{\textit{Case A}} \textit{ and}$$

$$\rho_\infty(\mathcal{L}(\boldsymbol{T}^*|\mathbf{X}), \mathcal{L}(\boldsymbol{T}|H_0)) \stackrel{p}{\longrightarrow} 0 \quad \textit{in } \textbf{\textit{Case B}}$$

*with the same interpretation in **Case B** as in Remark 2.1.*

Furthermore, these results imply that $\{H_0^{(\ell)} : \mathbf{c}_\ell'\boldsymbol{\mu} = 0, T_\ell, \ell = 1, \ldots, q\}$ constitute a joint testing family in the sense of Gabriel[10], because the joint distribution of an arbitrary selection of statistics $T_j, j \in \mathcal{J} \subseteq \{1, \ldots, q\}$ is completely specified under the hypothesis $H_0^{(\mathcal{J})} : \bigcap_{j\in\mathcal{J}}\{H_0^{(j)} : \boldsymbol{c}_j'\boldsymbol{\mu} = 0\}$ (asymptotically in **Case A**). Note that the simultaneous test procedure $\{H_0, \boldsymbol{T}, z_{1-\alpha}(max)\}$ is coherent, by construction, and therefore, $\{H_0, \boldsymbol{T}, z_{1-\alpha}(max)\}$ controls the FWER in the strong sense by Theorem 2 in[10].

3 The Proofs

Proof of Theorem 1. Since B.) is more complicated we only prove this part and note that part A.) follows similarly to the verification of the finite-dimensional (fidi) convergence below. Note, that due to the centring with $\sqrt{N}\boldsymbol{C}\boldsymbol{\mu}$ we may assume without restriction that $\boldsymbol{\mu} = \mathbf{0}$ holds. Moreover, since $\overline{\boldsymbol{Y}}_{1\cdot}$ and $\overline{\boldsymbol{Y}}_{2\cdot}$ are independent we can treat them separately. Consider $\overline{\boldsymbol{Y}}_{1\cdot}$. We first show that the Lindeberg condition is satisfied for the array $(Y_{1\ell k})_{k\leq n}$ for each fixed ℓ.

We therefore introduce $s_{n1\ell} := \sum_{k=1}^{n_1} Var(Y_{1\ell k}) = n_1 \cdot \boldsymbol{c}_\ell'\boldsymbol{\Sigma}_1\boldsymbol{c}_\ell$, which fulfills

$$s_{n1\ell}/n_1 \to v_{1,\ell\ell},$$

by assumption. Now suppose for a moment that $v_{1,\ell\ell} > 0$ holds. Then we have for all $\varepsilon > 0$: $P(|Y_{1\ell 1}| \geq \varepsilon s_{n1\ell}) \leq \varepsilon^{-2}\ Var(Y_{1\ell 1})/s_{n1\ell} = \varepsilon^{-2} n_1^{-1} \longrightarrow 0$. This implies the Lindeberg condition for the array $(Y_{1\ell k})_k$, since $\boldsymbol{c}_\ell' \boldsymbol{\Sigma}_1 \boldsymbol{c}_\ell \to v_{1,\ell\ell} > 0$ and

$$s_{n1\ell}^{-2} \sum_k \int Y_{1\ell k}^2 \mathbf{1}\{|Y_{1\ell k}| \geq \varepsilon s_{n1\ell}\} dP = \frac{n_1}{s_{n1\ell}} \int Y_{1\ell 1}^2 \mathbf{1}\{|Y_{1\ell 1}| \geq \varepsilon s_{n1\ell}\} dP \to 0$$

by Pratt's Lemma and the preceding considerations. In the case $v_{1,\ell\ell} = 0$ we may set $\tilde{Y}_{1\ell k}^2 = n_1 Y_{1\ell k}^2 / s_{n1\ell}$ (with $0/0 := 0$). In this case the first equality above becomes $\int \tilde{Y}_{1\ell k}^2 \mathbf{1}\{|\tilde{Y}_{1\ell k}| \geq \varepsilon \sqrt{n_1}\} dP$ which also converges to zero.
Now fix $r \in \mathbb{N}$ and set $\boldsymbol{Z}_{ik}^{(r)} = [(Y_{i\ell k})_{1\leq \ell \leq r}']'$, $1 \leq k \leq n_i, i = 1, 2$. Repeating the above steps for $\|\boldsymbol{Z}_{1k}^{(r)}\|$ shows that the r-dimensional array $\boldsymbol{Z}_{1k}^{(r)}$ satisfies the multivariate Lindeberg condition. Hence it follows that

$$\frac{1}{\sqrt{n_1}} \sum_{k=1}^{n_1} \boldsymbol{Z}_{1k}^{(r)} \xrightarrow{\mathcal{L}} N_r(\mathbf{0}, (v_{1,k\ell})_{k,\ell \leq r}).$$

Since the similar result also holds for the second sample (with the index 1 replaced by 2) and $v_{k\ell} = \lambda_1 v_{1,k\ell} + \lambda_2 v_{2,k\ell}$, we have

$$\sqrt{N} \Big(\frac{1}{n_1} \sum_{k=1}^{n_1} \boldsymbol{Z}_{1k}^{(r)} \pm \frac{1}{n_2} \sum_{k=1}^{n_2} \boldsymbol{Z}_{2k}^{(r)} \Big) \xrightarrow{\mathcal{L}} N_r(\mathbf{0}, (v_{k\ell})_{k,\ell \leq r}).$$

Finally, following Billingsley (1968, 29 f.) this proves part B.) since it suffices to show convergence of the finite-dimensional distributions. □

Proof of Lemma 1 Keeping the notation of the last proof (while still assuming $\boldsymbol{\mu} = \mathbf{0}$ w.l.o.g.) we recall that we have $s_{in\ell}/n_i \to v_{i,\ell\ell}$ for all ℓ and $i = 1, 2$. Since

$$\frac{n_i - 1}{n_i} \widehat{v}_{i,\ell\ell} = \frac{1}{n_i} \sum_{k=1}^{n_i} Y_{ik\ell}^2 - \Big(\frac{1}{n_i} \sum_{k=1}^{n_i} Y_{ik\ell}\Big)^2$$

and the latter part is uniformly negligible by Slutzky's Lemma and the proof of Theorem 1, it suffices to prove the result for $\widetilde{\boldsymbol{v}}_{i\ell\ell} := \frac{1}{n_i} \sum_{k=1}^{n_1} Y_{ik\ell}^2$ instead of $\widehat{v}_{i,\ell\ell}$. Since $(Y_{i\ell k})_k$ fulfills the Lindeberg condition and is centered, Raikov's Theorem, see Raikov (1938) or Janssen (2004, Lemma A.), implies convergence in probability

$$\frac{1}{n_i} \sum_{k=1}^{n_i} Y_{ik\ell}^2 \xrightarrow{p} v_{i,\ell\ell}$$

for every fixed ℓ in both the Cases A.) and B.). Note, that in Case B) we even have convergence in probability $(\widetilde{v}_{\ell\ell})_{\ell \in \mathbb{N}} \xrightarrow{p} (v_{\ell\ell})_{\ell \in \mathbb{N}}$ on $\mathbb{R}^{\mathbb{N}}$ by the subsequence principle for convergence in probability. Since sup is continuous on $\mathbb{R}^{\mathbb{N}}$ the assertion follows. □

□

Proof of Theorem 2 The result is a direct consequence of Lemma 1 in combination with Theorem 2. □

Proof of Theorem 3 As in the proof of Theorem 1 we only prove part B.) and note that A.) follows similarly. Moreover, with respect to Slutzky's Lemma we can treat the conditional convergences of $\boldsymbol{U}_N^*$ and $\widehat{\boldsymbol{D}}_V^*$ separately. Let us start with $\boldsymbol{U}_N^*$. Fix $r \in \mathbb{N}$ and consider the arrays $\boldsymbol{Z}_{ik}^{*(r)} = (Z_{i\ell k}^*)_{\ell \leq r}, 1 \leq k \leq n_i$ for $i = 1, 2$, where $\boldsymbol{Z}_{ik}^* = (Z_{i1k}^*, \dots, Z_{idk}^*)'$. Recall from the proof of Theorem 1 that the array $(Y_{i\ell k})'_{1 \leq \ell \leq r}]', 1 \leq k \leq n_i$ satisfies the multivariate Lindeberg condition. Hence it follows as in the proof of Lemma 1 that the following two convergences hold true

$$\frac{1}{n_i}\sum_{k=1}^{n_i}(Y_{i\ell k} - \overline{Y}_{i\ell\cdot})_{\ell \leq r}(Y_{i\ell k} - \overline{Y}_{i\ell\cdot})'_{\ell \leq r} \xrightarrow{p} (v_{i,k\ell})_{k,\ell \leq r},$$

$$\frac{1}{n}\max_{k \leq n} \|(Y_{\ell k} - \overline{Y}_{\ell\cdot})_{\ell \leq r}\|^2 \xrightarrow{p} 0.$$

Hence an application of Theorem A.1 in Beyersmann et al. (2013) shows that the conditional distribution of $\sqrt{n_i}(\overline{Z}_{i1\cdot}^*, \dots, \overline{Z}_{ir\cdot}^*)$ given the data converges weakly in probability to $N_r(\mathbf{0}_r, (v_{ik\ell})_{k,\ell \leq r})$. Again applying Billingsley (1968, 29 f.) this proves that $\sqrt{n_i}\overline{Z}_{i\cdot}^*$ is weakly asymptotically $N_\infty(\mathbf{0}_\infty, \boldsymbol{V}_i)$ in probability given the data. Hence it follows that $\sqrt{N}U_N^*$ given the data converges weakly in probability to $N_\infty(\mathbf{0}_\infty, \boldsymbol{V}_\infty)$ and it only remains to investigate $\widehat{\boldsymbol{D}}^*$. To this end we will again apply Raikov's Theorem in its multivariate version; but now point-wise (i.e. given the data). Note that for fixed observations $\boldsymbol{Z}_{ik}^*, 1 \leq k \leq n_i$ defines an array of row-wise i.i.d. random variables for $i = 1, 2$. From the proof of Theorem A1 in Beyersmann et al. (2013) it follows that the array $\boldsymbol{Z}_{ik}^{*(r)}$ fulfills the Lindeberg condition in probability. Hence a two-fold application of the subsequence principle for convergence in probability shows that

$$\frac{1}{n_i}\sum_{k=1}^{n_i}(Z_{i\ell k} - \overline{Z}_{i\ell\cdot})_{\ell \leq r}(Z_{i\ell k} - \overline{Z}_{i\ell\cdot})'_{\ell \leq r}$$

converges in probability to the matrix $(v_{k\ell})_{k\ell \leq r}$. This proves that $\widehat{\boldsymbol{D}}^*$ converges in probability to $diag\{v_{\ell\ell} : \ell \in \mathbb{N}\}$ and the result follows. □

4 Additional Information for Protein Abundance Data

Table 1. Means and variances of the protein abundance data for each protein × region × group combination. The meaning of the dimension is listed below (1.1).

	Means		Empirical Variances	
Dimension	Group 1	Group 2	Group 1	Group 2
1	16.83	16.44	2.30	6.22
2	17.46	16.95	0.88	4.14
3	15.83	13.70	1.56	9.62
4	15.26	14.26	3.52	4.87
5	16.13	15.10	2.46	6.94
6	17.36	16.18	3.75	5.30
7	16.99	17.60	0.74	0.33
8	15.74	16.82	0.84	0.49
9	17.03	17.27	0.68	0.06
10	16.12	16.32	0.31	0.21
11	17.67	17.83	0.52	0.17
12	18.49	18.57	0.07	0.05
13	18.38	17.79	0.35	0.83
14	18.67	18.61	0.23	0.34
15	13.39	13.48	5.80	4.89
16	14.64	14.92	0.91	1.09
17	17.69	17.31	0.11	1.19
18	18.26	18.11	0.22	0.71
19	15.09	15.95	1.24	2.21
20	15.47	16.85	1.47	3.76
21	16.82	16.58	0.48	0.48
22	14.61	15.08	0.43	0.47
23	17.15	16.72	0.34	3.96
24	18.44	18.17	0.15	0.76
25	18.62	18.78	0.04	0.01
26	18.29	18.60	0.10	0.05
27	15.19	16.73	3.41	1.98
28	13.61	14.98	5.01	2.65
29	18.06	18.08	0.50	0.35
30	16.66	16.94	2.46	2.44
31	17.50	17.10	0.37	0.08
32	17.65	17.58	0.23	0.12
33	16.28	15.68	0.52	0.93
34	15.78	15.27	0.50	0.45
35	17.43	17.57	0.44	0.31
36	18.68	18.38	0.03	0.16

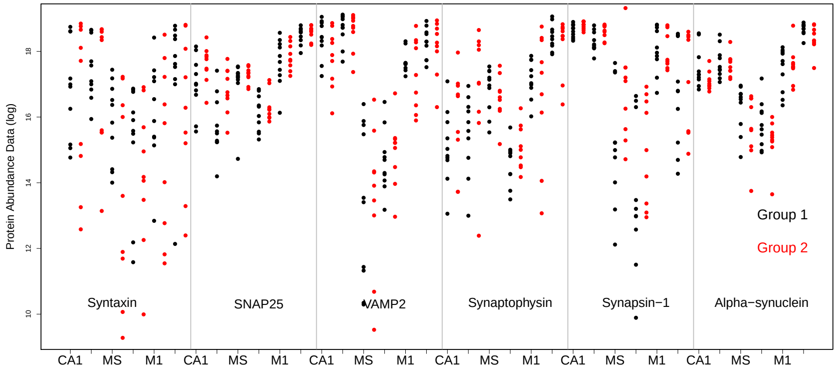

Figure 1. Dotplots (log) of the Protein Abundance trial.

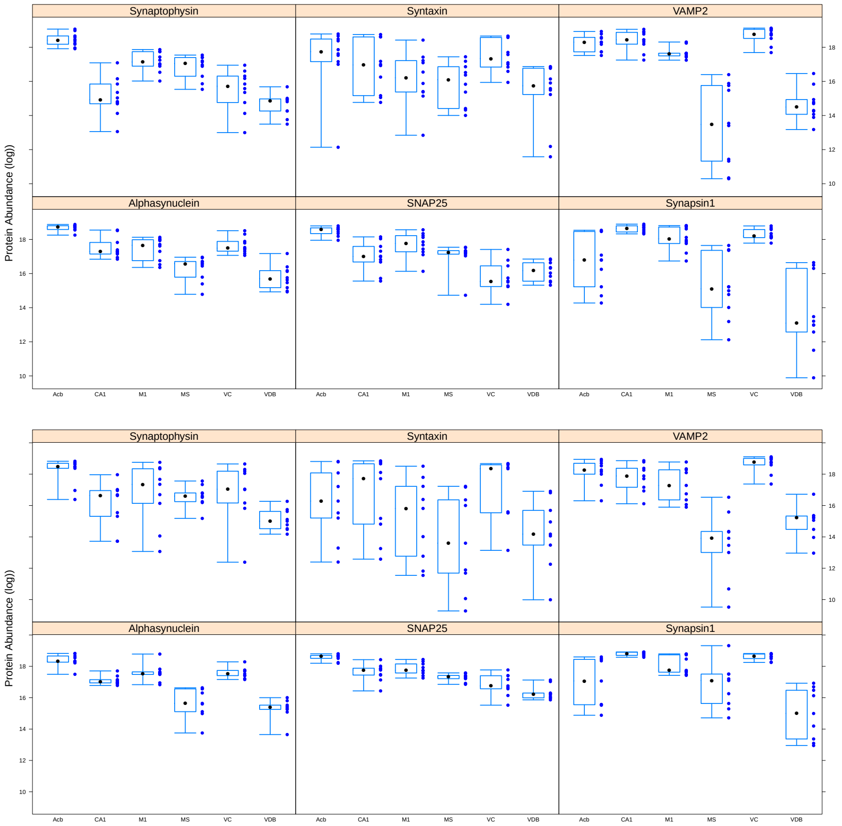

Figure 2. Boxplots of the protein abundance data (log-scale). Here, data of group 1 are in the upper panel.

5 Simulation Results

5.1 Type-1 error simulation results

Due to the abundance of possible factorial designs and hypotheses, two-way designs with varying dimension $d \in \{2, 4, \ldots, 150\}$ will be simulated and the hypothesis H_0 : $\boldsymbol{P}_d(\boldsymbol{\mu}_1 - \boldsymbol{\mu}_2) = \mathbf{0}$ of *no interaction effect* will be tested at 5% level of significance. Data was generated from model

$$\boldsymbol{X}_{ik} \sim \boldsymbol{F}_i(\boldsymbol{\mu}_0, \boldsymbol{\Sigma}_i) + \boldsymbol{\mu}_i,\ i = 1, 2,\ k = 1, \ldots, n_i, \tag{5.7}$$

where $\boldsymbol{F}_i(\boldsymbol{\mu}_0, \boldsymbol{\Sigma}_i)$ represents a multivariate distribution with expectation vector $\boldsymbol{\mu}_0$, correlation matrix $\boldsymbol{\Sigma}_i$ and location shifts $\boldsymbol{\mu}_i$. As representative marginal data distributions, we selected three differently tailed symmetric distributions (normal, logistic, T_3) and three skewed distributions (ranging from mildly to very skewed) (χ^2_7, χ^2_{15}, exponential) each with sample sizes $n_i \in \{10, 20\}$. A major assessment criteria of the quality of the proposed approximations is the impact of both the chosen contrast as well as the dependency structures of the data—especially when data has different covariance matrices and thus covering a typical Behrens-Fisher situation. Here, we used normal copulas in order to generate rather complex dependency structures of the repeated measurements using the R-package *copula*[11]. The different allocations of the correlation matrices used in the simulation studies are summarized in Table 2.

Table 2. Different correlation matrices used in the simulation study.

**Setting 1**:	$\boldsymbol{\Sigma}_1 = (\sigma_{1,ij}) = 0.6^{\lvert i-j\rvert}$	$\boldsymbol{\Sigma}_2 = (\sigma_{2,ij}) = 0.6^{\lvert i-j\rvert}$
**Setting 2**:	$\boldsymbol{\Sigma}_1 = (\sigma_{1,ij}) = 0.6^{\lvert i-j\rvert/(d-1)}$	$\boldsymbol{\Sigma}_2 = (\sigma_{2,ij}) = 0.6^{\lvert i-j\rvert}$
**Setting 3**:	$\boldsymbol{\Sigma}_1 = (\sigma_{1,ij}) = 1 - \lvert i-j\rvert/d$	$\boldsymbol{\Sigma}_2 = (\sigma_{2,ij}) = 0.6^{\lvert i-j\rvert/(d-1)}$
**Setting 4**:	$\boldsymbol{\Sigma}_1 = \boldsymbol{I}_d + 0.5 \cdot (\boldsymbol{J}_d - \boldsymbol{I}_d)$	$\boldsymbol{\Sigma}_2 = \boldsymbol{I}_d + 0.25 \cdot (\boldsymbol{J}_d - \boldsymbol{I}_d)$.

In Setting 1, both correlation matrices $\boldsymbol{\Sigma}_1$ and $\boldsymbol{\Sigma}_2$ are identical and represent an autoregressive structure. In Settings 2 and 3, the covariance matrices $\boldsymbol{\Sigma}_1$ and $\boldsymbol{\Sigma}_2$ have different off-diagonal elements models, whereas an autoregressive structure depending on the dimension d is modeled by $\boldsymbol{\Sigma}_1$ in Setting 2, and a linearly decreasing (symmetric) Toeplitz structure is covered by $\boldsymbol{\Sigma}_1$ in Setting 3 (see Table 2), see[7] for similar choices. For a detailed overview of copulas we refer to Nelsen (2007)[12] or Marozzi (2015)[13].

All these four settings will be simulated for all four sample sizes ($n_i \in \{10, 20\}$), dimensions ($d \in \{2, 4, \ldots, 150\}$) and distributional configurations as described above. The type-1 error simulation results obtained under Setting 1 are displayed in Figure 3.

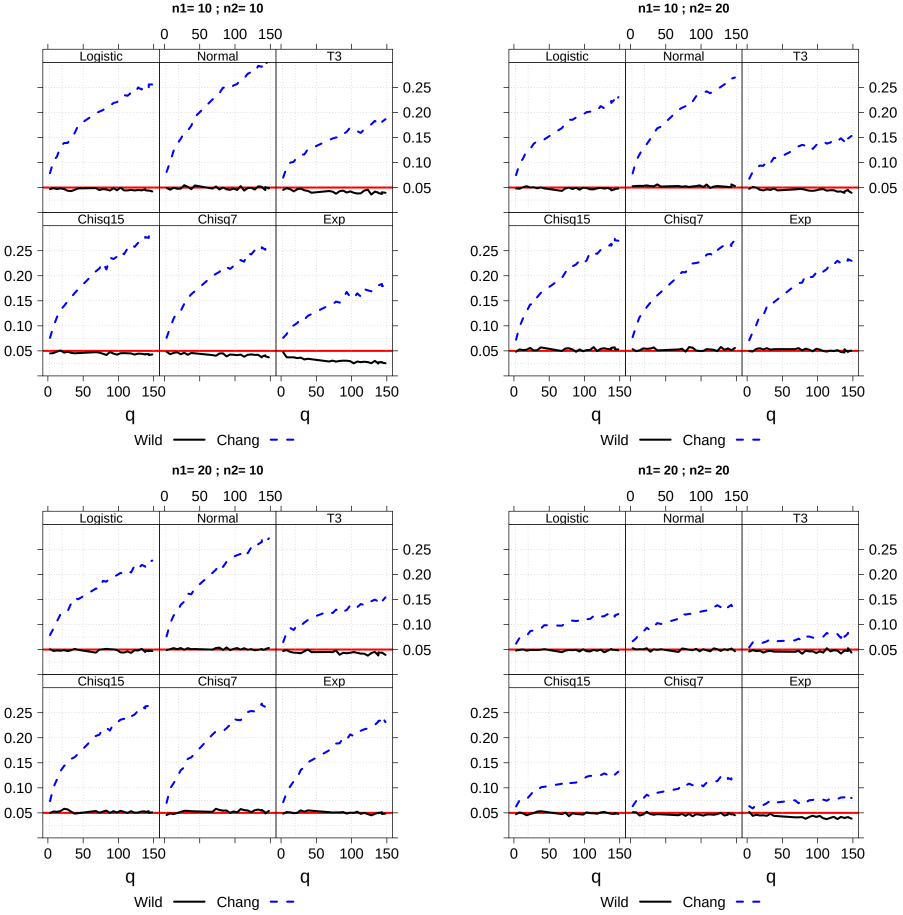

Figure 3. Type-1 error ($\alpha = 5\%$) simulation results of the Wild-bootstrap randomization test (*Wild*) and simulation-based test (*Chang*). Data have covariance matrices as described in Setting 1 in Table 2.

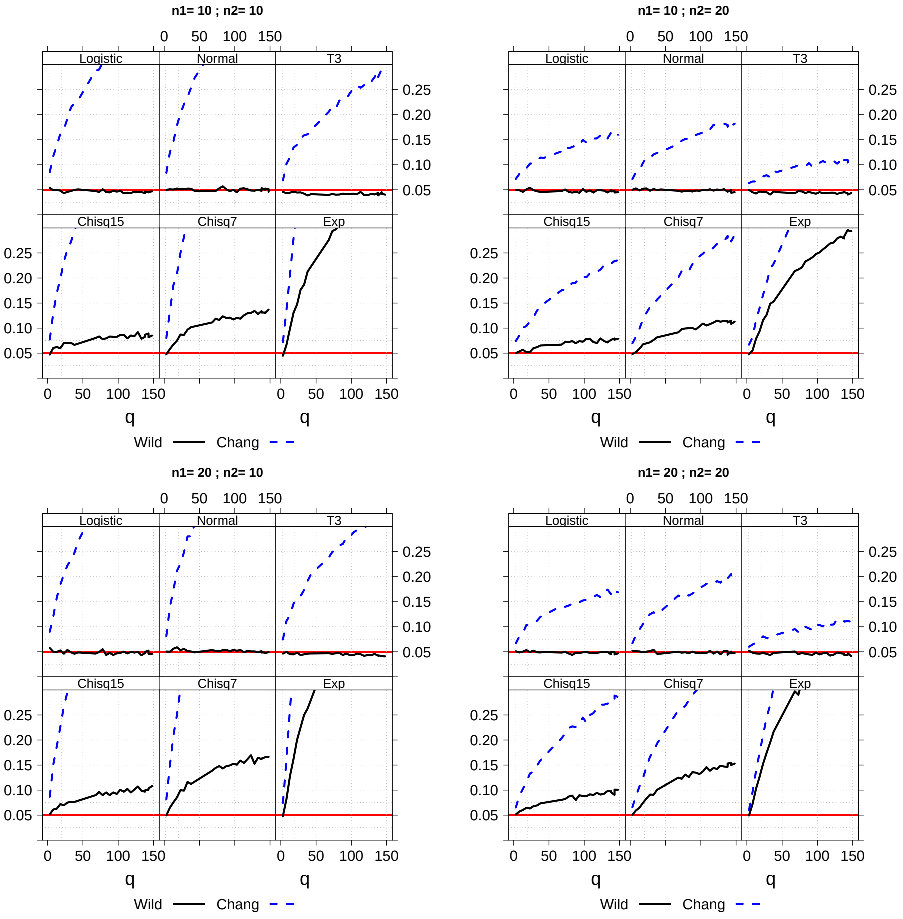

Figure 4. Type-1 error ($\alpha = 5\%$) simulation results of the Wild-bootstrap randomization test (*Wild*) and simulation-based test (*Chang*). Data have covariance matrices as described in Setting 2 in Table 2.

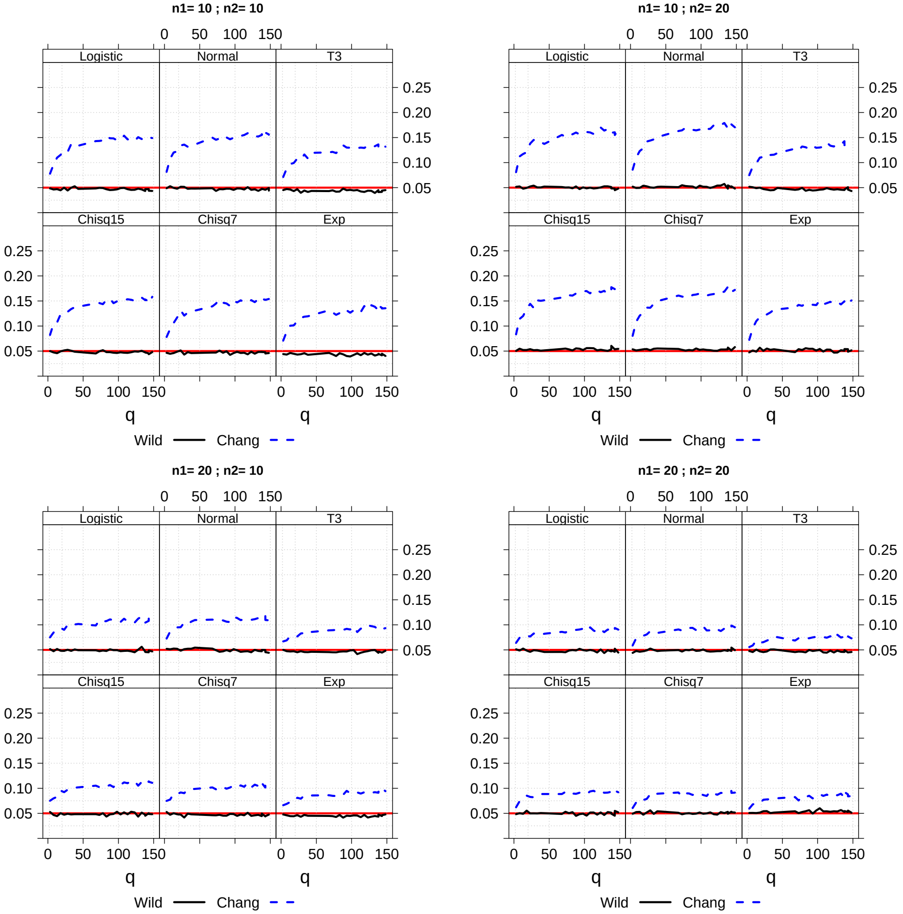

Figure 5. Type-1 error ($\alpha = 5\%$) simulation results of the Wild-bootstrap randomization test (*Wild*) and simulation-based test (*Chang*). Data have covariance matrices as described in Setting 3 in Table 2.

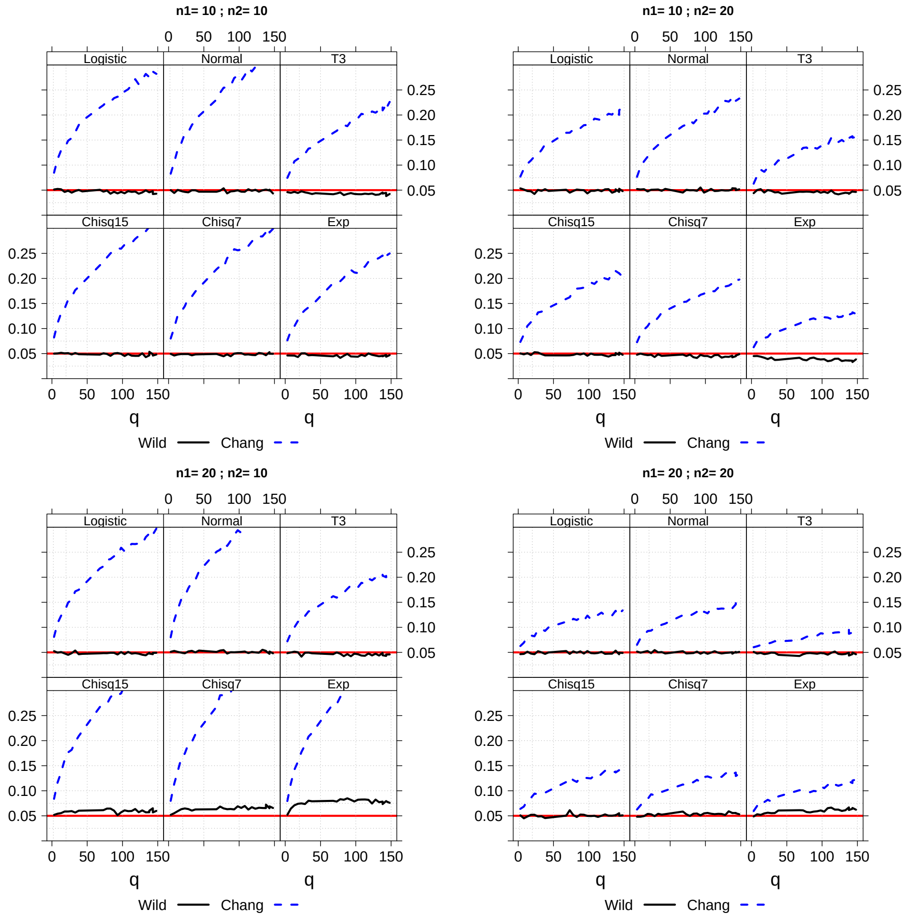

Figure 6. Type-1 error ($\alpha = 5\%$) simulation results of the Wild-bootstrap randomization test (*Wild*) and simulation-based test (*Chang*). Data have covariance matrices as described in Setting 4 in Table 2.

5.2 Power simulation results

The any-pairs power curves are displayed in Figure 7 (small sample sizes) and in Figure 8 (large sample sizes) . All-pairs power curves are provided in Figure 9 (small sample sizes) and Figure 10 (large sample sizes) and in F, respectively.

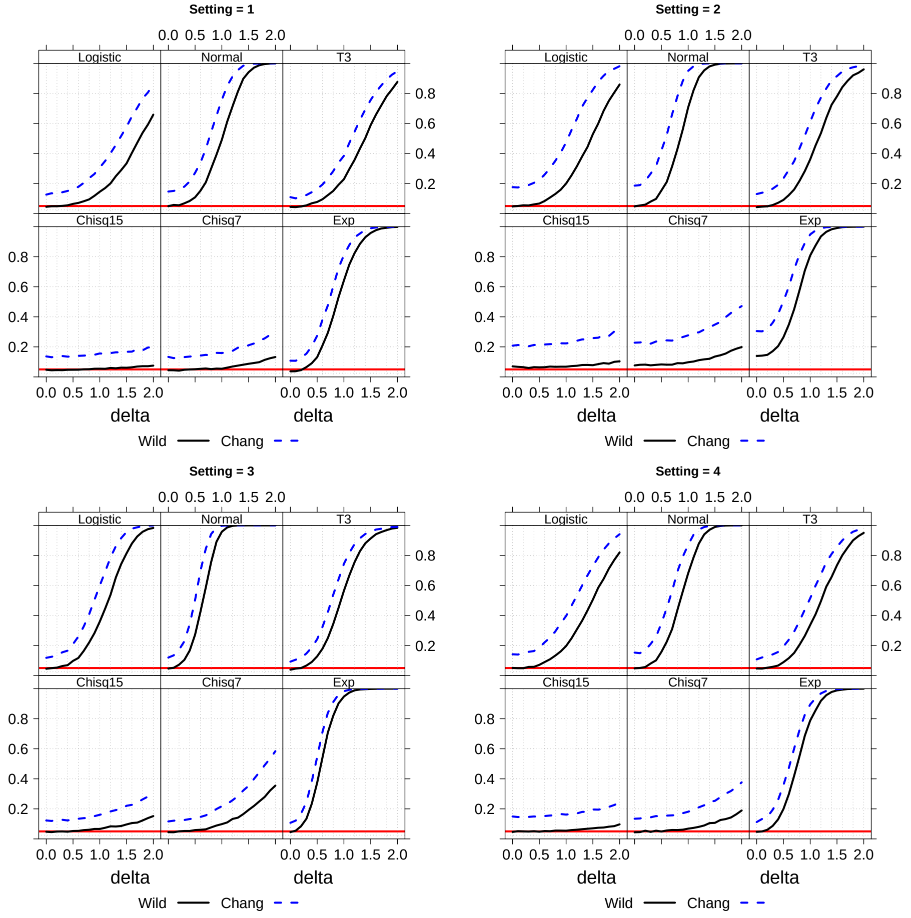

Figure 7. Any-pairs power ($\alpha = 5\%$) simulation results of the Wild-bootstrap randomization test (*Wild*) and simulation-based test (*Chang*) with small ($n_i = 10$) sample sizes. Data have covariance matrices as described in Table 2.

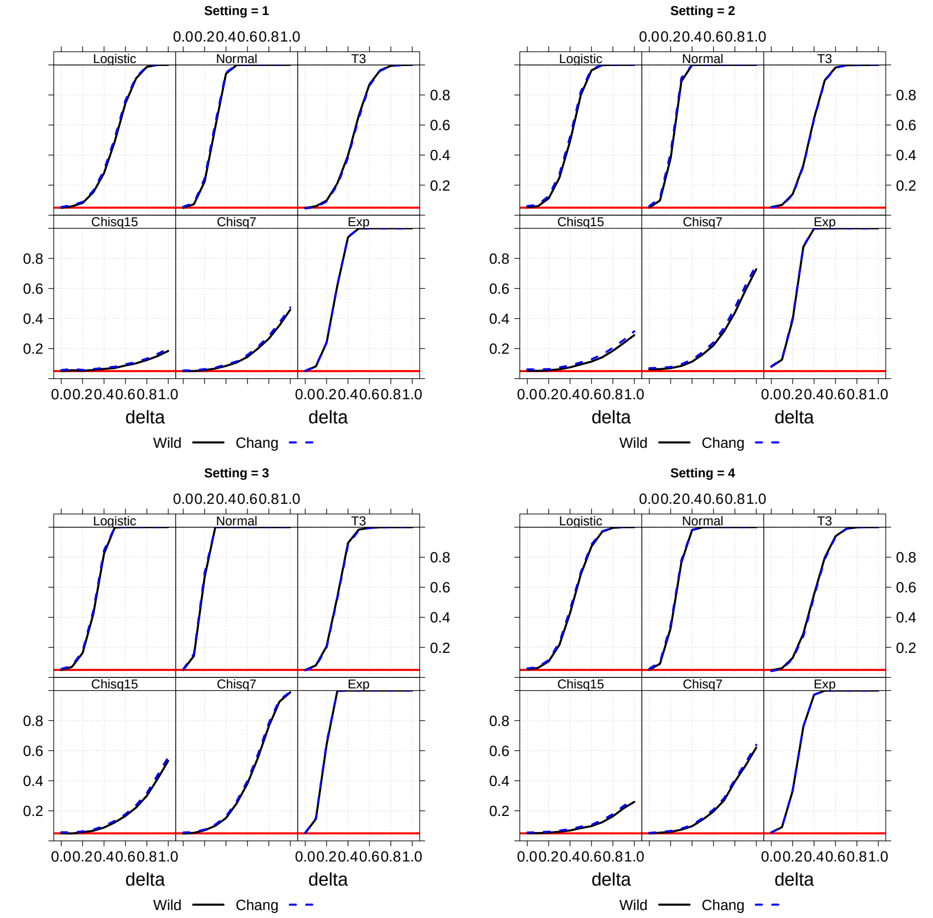

Figure 8. Any-pairs power ($\alpha = 5\%$) simulation results of the Wild-bootstrap randomization test (*Wild*) and simulation-based test $\boldsymbol{T}$ (*Chang*) with large ($n_i = 100$) sample sizes. Data have covariance matrices as described in Table 2.

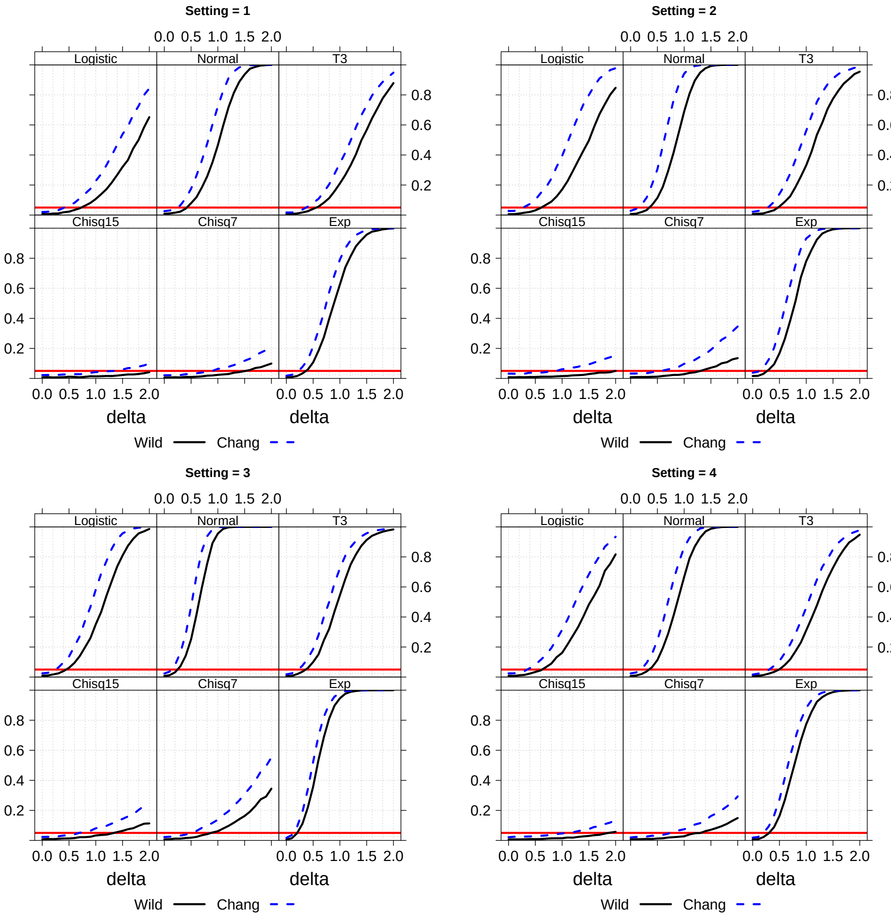

Figure 9. All-pairs power ($\alpha = 5\%$) simulation results of the Wild-bootstrap randomization test (*Wild*) and simulation-based test $\boldsymbol{T}$ (*Chang*) with small ($n_i = 10$) sample sizes. Data have covariance matrices as described in Table 2.

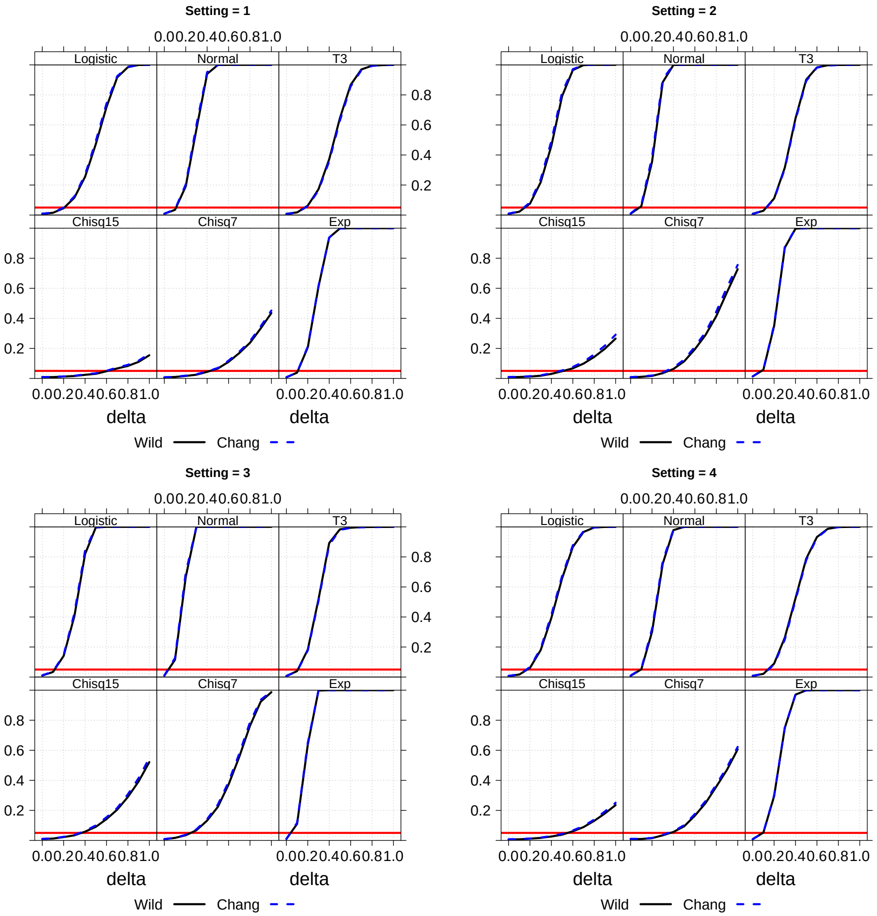

Figure 10. All-pairs power ($\alpha = 5\%$) simulation results of the Wild-bootstrap randomization test (*Wild*) and simulation-based test (*Chang*) with large ($n_i = 100$) sample sizes. Data have covariance matrices as described in Table 2.

6 Particular Designs and Hypotheses

In order to demonstrate the application of the procedures, we consider some frequently occuring designs. Our general apporach includes the so-called (simple) split-splot repeated measures design[14] where it is assumed that $\boldsymbol{X}_{ik} = (X_{i1k}, \ldots, X_{idk})' \sim N(\boldsymbol{\mu}_i, \boldsymbol{\Sigma}_i),\ i = 1, 2$. In our more general model we drop the assumption of the normal distribution and require only that the $\boldsymbol{X}_i$ are i.i.d. with $E(\boldsymbol{X}_i) = \boldsymbol{\mu}_i$ and $Cov(\boldsymbol{X}_i) = \boldsymbol{\Sigma}_i > 0$. Moreover, we even allow that the dimension d can be much larger than the sample size N. Note that also the case of unequal covariance matrices is included in this set-up, thus covering the generalized multivariate Behrens-Fisher problem. Even under the assumption of normality it is well known that the assumption of equal covariance matrices is crucial for classical multivariate inference procedures. In the high-dimensional case it has been demonstrated by[15] that this assumption is even more crucial. To take into account the problem of unequal covariance matrices in the multivariate high-dimensional two-sample set-up several techniques have been developed recently,[7,8,16,17]. Although our approach can also be used for such a multivariate testing problem, it is not the major aim of the present paper, which is developing multiple testing procedures for repeated measurements, where also hypotheses about the components of the vectors are of interest.

As another example, our general approach particularly covers models with random subject effects $c \cdot S_k,\ k = 1, \ldots, n_i$, where the S_k are i.i.d. random variables with $E(S_k) = 0$ and $Var(S_k) = 1$. Here, c is an unknown scaling factor to model the variance of the unknown random subject effect. The corresponding semiparametric mixed effects model is written as

$$\boldsymbol{X}_{ik} = \boldsymbol{\mu}_i + cS_k\boldsymbol{1}_d + \boldsymbol{\epsilon}_{ik},$$

where $\boldsymbol{\epsilon}_{ik} = (\boldsymbol{\epsilon}_{i1k}, \ldots, \boldsymbol{\epsilon}_{idk})'$ are i.i.d. error terms with $E(\boldsymbol{\epsilon}_{i1}) = \boldsymbol{0}$ and $Cov(\boldsymbol{\epsilon}_{i1}) = \boldsymbol{S}_i > 0$ which are independent of S_k. Thus, $Cov(X_{i1}) = \boldsymbol{\Sigma}_i = \boldsymbol{S}_i + c^2\boldsymbol{J}_d$ with $\boldsymbol{J}_d = \boldsymbol{1}_d\boldsymbol{1}_d'$. Common covariance choices of $\boldsymbol{S}_i$ as the identity, compound symmetry or an autoregressive structure are special cases and fulfill the Assumptions.

Factorial structures on the repeated measures are introduced by splitting the index $j = 1, \ldots d$ into subindices. For example, a setting with two factors is achieved by setting $d = ab$ and denoting the levels of factor A with $j_1 = 1, \ldots, a$ and the levels of factor B with $j_2 = 1, \ldots, b$. Such designs occur frequently in biological or medical trials when the observations are repeatedly taken over time (e.g., factor A) on paired organs or different parts of the same subject (e.g., factor B) under two different treatments, $i = 1, 2$. For a practical occurrence, see e.g., the sleep laboratory trial of[18] as well as the postoperative edema study on p. 18 in[19]. Using the notation introduced in Section 1, the hypotheses of parallel time profiles for the different parts of the subject under different treatments (factor T) is written as

$$H_0(ABT) : (\boldsymbol{m}_d \otimes \boldsymbol{P}_a \otimes \boldsymbol{P}_b)\boldsymbol{\mu} = \boldsymbol{0}. \qquad \text{(three-fold ABT-interaction)}$$

Here, $\boldsymbol{\mu}_i = (\boldsymbol{\mu}_{i1}', \ldots, \boldsymbol{\mu}_{ia}')$, where $\boldsymbol{\mu}_{ij_1} = (\boldsymbol{\mu}_{ij_11}, \ldots, \boldsymbol{\mu}_{ij_1b})'$ is structured according to the hypothesis matrix $\boldsymbol{m}_d \otimes \boldsymbol{P}_a \otimes \boldsymbol{P}_b$. As another example, many-to-one comparisons

for different treatment effects on the time points with respect to the baseline time averaged over the different parts of the subjects can be written as

$$H_0(AT) : (\boldsymbol{m}_d \otimes \boldsymbol{M}_a \otimes \tfrac{1}{b}\boldsymbol{J}_b)\boldsymbol{\mu} = \boldsymbol{0}. \qquad \text{(two-fold AT-interaction)}$$

For other hypotheses in this design, see e.g. Brunner et al. (2012, Section 4.1).

7 Simulation Code

```
library(multcomp)
library(copula)

simu_CI2<-function(n1,n2,d,Sigma1,Sigma2,nsim, nboot, Distribution, Setting){
  #-------------------------Useful Matrices------------------------#
  ###alpha=0.05
        WRade=WCheng=c()

  Pd=diag(d)-1/d
  nc<-nrow(Pd)
  N<-n1+n2
        #--------------------------Bootstrap Weights--------------------------------#
  WR<-matrix(rbinom(N*nboot,1,1/2)*2-1, nrow=nboot, ncol=N)
  WR1<-WR[,1:n1]
  WR2<-WR[,(n1+1):N]

  #-----------------------Matrices for Means----------------------------------#
  WRM1 <- 1/n1*WR1
  WRM2 <- 1/n2*WR2
  Pn1 <- diag(n1) - 1/n1
  Pn2 <- diag(n2) - 1/n2
  #----------------------Matrices for Variances-------------------------------#
  WR12 <- WR1^2
  WR22 <- WR2^2
  WRV1 <- 1/(n1-1)*WR12
  WRV2 <- 1/(n2-1)*WR22
  #-----------Compute Parameters for Copula Generation -----------------------#
  Rho1= cov2cor(Sigma1)
  URho1= upper.tri(Rho1)
  rvec1=NULL
   Rho2= cov2cor(Sigma2)
  URho2= upper.tri(Rho2)
  rvec2=NULL
  for(rv1 in 1:d){
  for(rv2 in 1:d){
  if(URho1[rv1,rv2]){rvec1=c(rvec1,Rho1[rv1,rv2])}
  if(URho2[rv1,rv2]){rvec2=c(rvec2,Rho2[rv1,rv2])}}}
  #--------------------------Data Generation-----------------------------------#
  if(Distribution=="Normal"){
        cop1 <- mvdc( copula=normalCopula(rvec1, dim = d, dispstr = "un"),
          margins=rep("norm",d),
          paramMargins=lapply(1:d,function(arg){list(mean=0,sd=1)}))

  cop2 <- mvdc( copula=normalCopula(rvec2, dim = d, dispstr = "un"),
          margins=rep("norm",d),
          paramMargins=lapply(1:d,function(arg){list(mean=0,sd=1)}))
    x1 <- rMvdc(n1*nsim,cop1) %*%Pd
    x2 <- rMvdc(n2*nsim,cop2)%*%Pd}

  if(Distribution=="T3"){
  cop1 <- mvdc( copula=normalCopula(rvec1, dim = d, dispstr = "un"),
          margins=rep("t",d),
          paramMargins=lapply(1:d,function(arg){list(df=3)}))

        cop2 <- mvdc( copula=normalCopula(rvec2, dim = d, dispstr = "un"),
          margins=rep("t",d),
          paramMargins=lapply(1:d,function(arg){list(df=3)}))
    x1 <- rMvdc(n1*nsim,cop1) %*%Pd
    x2 <- rMvdc(n2*nsim,cop2)%*%Pd}

  if(Distribution=="Logistic"){
   cop1 <- mvdc( copula=normalCopula(rvec1, dim = d, dispstr = "un"),
           margins=rep("logis",d),
           paramMargins=lapply(1:d,function(arg){list(location = 0, scale = 1)}))
        cop2 <- mvdc( copula=normalCopula(rvec2, dim = d, dispstr = "un"),
            margins=rep("logis",d),
            paramMargins=lapply(1:d,function(arg){list(location = 0, scale = 1)}))
    x1 <- rMvdc(n1*nsim,cop1) %*%Pd
    x2 <- rMvdc(n2*nsim,cop2)%*%Pd}
                if(Distribution=="Exp"){
   cop1 <- mvdc(copula=normalCopula(rvec1, dim = d, dispstr = "un"),
```

```
          margins=rep("exp",d),
          paramMargins=lapply(1:d,function(arg){list(rate=1)}))
   cop2 <- mvdc( copula=normalCopula(rvec2, dim = d, dispstr = "un"),
          margins=rep("exp",d),
          paramMargins=lapply(1:d,function(arg){list(rate=1)}))
    x1 <- rMvdc(n1*nsim,cop1)%*%Pd
    x2 <- rMvdc(n2*nsim,cop2)%*%Pd}
  if(Distribution=="Chisq7"){
    cop1 <- mvdc( copula=normalCopula(rvec1, dim = d, dispstr = "un"),
            margins=rep("chisq",d),
            paramMargins=lapply(1:d,function(arg){list(df=7)}))
    cop2 <- mvdc(copula=normalCopula(rvec2, dim = d, dispstr = "un"),
            margins=rep("chisq",d),
            paramMargins=lapply(1:d,function(arg){list(df=7)}))
    x1 <- rMvdc(n1*nsim,cop1)%*%Pd
    x2 <- rMvdc(n2*nsim,cop2)%*%Pd}

   if(Distribution=="Chisq15"){
    cop1 <- mvdc( copula=normalCopula(rvec1, dim = d, dispstr = "un"),
            margins=rep("chisq",d),
            paramMargins=lapply(1:d,function(arg){list(df=15)}))

    cop2 <- mvdc(copula=normalCopula(rvec2, dim = d, dispstr = "un"),
            margins=rep("chisq",d),
            paramMargins=lapply(1:d,function(arg){list(df=15)}))
    x1 <- rMvdc(n1*nsim,cop1) %*%Pd
    x2 <-  rMvdc(n2*nsim,cop2)%*%Pd}

#-----------------------Center the Data Vectors--------------------------------#

  x1Z<-matrix(0,nrow=n1*nsim,ncol=nc)
  x2Z<-matrix(0,nrow=n2*nsim,ncol=nc)
  num1<-1:n1
  num2<-1:n2
  for (j in 0:(nsim-1)){
    s11<-num1+j*n1
    s12<-num2+j*n2
    pl1<-x1[s11,]
    pl2<-x2[s12,]
    plZ1<-Pn1%*%pl1
    plZ2<-Pn2%*%pl2
    x1Z[s11,]<-plZ1
    x2Z[s12,]<-plZ2
  }

  x12 <- x1^2
  x22 <- x2^2
  x1Z2<-x1Z^2
  x2Z2<-x2Z^2
  #-------------------Begin of Simulation Loop---------------------------------#
  for (i in 0:(nsim-1)){
    s1<-num1+i*n1
    s2<-num2+i*n2
    pl1<-x1[s1,]
    pl12 <- x12[s1,]
    pl1Z <- x1Z[s1,]
    pl1Z2 <-x1Z2[s1,]
    mx1 <- colMeans(pl1)
    vx1 <- (colSums(pl12)-n1*mx1^2)/((n1-1))
    pl2<-x2[s2,]
    pl22 <- x22[s2,]
    pl2Z <- x2Z[s2,]
    pl2Z2 <-x2Z2[s2,]
    mx2 <- colMeans(pl2)
    vx2 <- (colSums(pl22)-n2*mx2^2)/((n2-1))
    mx <- (mx1-mx2)
    vx<-(vx1/n1+ vx2/n2)

    #--------------------------Original Statistic T0---------------------------#
    Torig <- mx/sqrt(vx)
    Tmax  <- max(abs(Torig))
    ######################################################################
    #-----------------------------Wild- Bootstrap-------------------------#
    ######################################################################

    #----------------------------Rademacher Weights--------------------------#

    MZR1 <- WRM1%*%pl1Z
    VZR1 <- (WRV1%*%pl1Z2-n1*MZR1^2/(n1-1))/(n1)

    MZR2 <- WRM2%*%pl2Z
    VZR2 <- (WRV2%*%pl2Z2-n2*MZR2^2/(n2-1))/(n2)
    TR0  <- (MZR1-MZR2 ) / sqrt(VZR1+VZR2)
    TR   <- abs(TR0)
    WRade[i+1]<-(mean(((rowSums(TR>=Tmax))>=1))<0.05)
    #----------- Implement the Cheng Method ------------------------#
```

```
    SBoot=rmvnorm(n=nboot,sigma=cov2cor(var(pl1)/n1 +var(pl2)/n2),method="svd")
    TSB <- abs(SBoot)

    WCheng[i+1]<-(mean(((rowSums(TSB>=Tmax))>=1))<0.05)

   #--------------------Ende of Simulation Loop ------------------------------#
  }

  Result<-data.frame(
    nsim=nsim, nboot=nboot,d=d,n1=n1,n2=n2,
    WRade=mean(WRade),
    Cheng = mean(WCheng),
    Distribution=Distribution,
    Setting=Setting)
   print(Result)

 write.table(Result,file="Simu_Typ1_Copula_Final.txt",eol="\r\n",
  row.names=FALSE,col.names=FALSE,quote=FALSE,append=TRUE)

}



################################################################################


AR1<-function(d){
  si<-matrix(0,ncol=d,nrow=d)
  for (i in 1:d){ for(j in 1:d) {
    si[i,j]<-(0.6)^((abs(i-j)) )
  }}
  si}

AR2<-function(d){
  si<-matrix(0,ncol=d,nrow=d)
  for (i in 1:d){ for(j in 1:d) {
    si[i,j]<-(0.6)^((abs(i-j)/(d-1)) )
  }}
  si}

TO=function(d){
  si<-matrix(0,ncol=d,nrow=d)
  for (i in 1:d){ for(j in 1:d) {
    si[i,j]<-1-abs(i-j)/d
  }}
  si}

CS1=function(d){
  diag(d)+0.5*(1-diag(d))}

CS2=function(d){
  diag(d)*2+0.5*(1-diag(d))}


n1<-c(10,20)
n2<-c(10,20)
d <- seq(33,150,5)
Distribution <- c("Normal","Logistic", "T3", "Chisq7", "Chisq15", "Exp")

  nsims=10000
  nboots=1000

for(p3 in 1:length(d)){
  for(p4 in 1:length(Distribution)){

    simu_CI2(10,10,d[p3],AR1(d[p3]),AR1(d[p3]),nsims,nboots,Distribution[p4],1)
    simu_CI2(10,10,d[p3],AR2(d[p3]),AR1(d[p3]),nsims,nboots,Distribution[p4],2)
    simu_CI2(10,10,d[p3],TO(d[p3]),AR2(d[p3]),nsims,nboots,Distribution[p4],3)
    simu_CI2(10,10,d[p3],CS1(d[p3]),CS2(d[p3]),nsims,nboots,Distribution[p4],4)

    simu_CI2(10,20,d[p3],AR1(d[p3]),AR1(d[p3]),nsims,nboots,Distribution[p4],1)
    simu_CI2(10,20,d[p3],AR2(d[p3]),AR1(d[p3]),nsims,nboots,Distribution[p4],2)
    simu_CI2(10,20,d[p3],TO(d[p3]),AR2(d[p3]),nsims,nboots,Distribution[p4],3)
    simu_CI2(10,20,d[p3],CS1(d[p3]),CS2(d[p3]),nsims,nboots,Distribution[p4],4)

    simu_CI2(20,10,d[p3],AR1(d[p3]),AR1(d[p3]),nsims,nboots,Distribution[p4],1)
    simu_CI2(20,10,d[p3],AR2(d[p3]),AR1(d[p3]),nsims,nboots,Distribution[p4],2)
    simu_CI2(20,10,d[p3],TO(d[p3]),AR2(d[p3]),nsims,nboots,Distribution[p4],3)
    simu_CI2(20,10,d[p3],CS1(d[p3]),CS2(d[p3]),nsims,nboots,Distribution[p4],4)

    simu_CI2(20,20,d[p3],AR1(d[p3]),AR1(d[p3]),nsims,nboots,Distribution[p4],1)
    simu_CI2(20,20,d[p3],AR2(d[p3]),AR1(d[p3]),nsims,nboots,Distribution[p4],2)
    simu_CI2(20,20,d[p3],TO(d[p3]),AR2(d[p3]),nsims,nboots,Distribution[p4],3)
    simu_CI2(20,20,d[p3],CS1(d[p3]),CS2(d[p3]),nsims,nboots,Distribution[p4],4)
    }}
```

References

1. Liu W, Ah-Kine P, Bretz F et al. Exact simultaneous confidence intervals for a finite set of contrasts of three, four or five generally correlated normal means. *Computational Statistics & Data Analysis* 2013; 57(1): 141–148.
2. Hasler M. Multiple contrasts for repeated measures. *The International Journal of Biostatistics* 2013; 9(1): 49–61.
3. Hasler M. Multiple contrast tests for multiple endpoints in the presence of heteroscedasticity. *The International Journal of Biostatistics* 2014; 10(1): 17–28.
4. Hasler M and Böhlendorf K. Multiple comparisons for multiple endpoints in agricultural experiments. *Journal of Agricultural, Biological, and Environmental Statistics* 2013; 18(4): 1–16.
5. Hothorn T, Bretz F and Westfall P. Simultaneous inference in general parametric models. *Biometrical Journal* 2008; 50(3): 346 –363.
6. Hothorn T, Bretz F, Westfall P et al. *multcomp: Simultaneous Inference in General Parametric Models*. R Foundation for Statistical Computing, Wien, Austria, 2017. URL http://www.R-project.org.
7. Pauly M, Ellenberger D and Brunner E. Analysis of high-dimensional one group repeated measures designs. *Statistics* 2015; 49(6): 1243–1261.
8. Chen SX and Qin YL. A two-sample test for high-dimensional data with applications to gene-set testing. *The Annals of Statistics* 2010; 38(2): 808–835.
9. Chang J, Zheng C, Zhou WX et al. Simulation-based hypothesis testing of high dimensional means under covariance heterogeneity. *Biometrics* 2017; 73(4): 1300–1310.
10. Gabriel KR. Simultaneous test procedures–some theory of multiple comparisons. *The Annals of Mathematical Statistics* 1969; 4(1): 224–250.
11. Yan J et al. Enjoy the joy of copulas: with a package copula. *Journal of Statistical Software* 2007; 21(4): 1–21.
12. Nelsen RB. *An introduction to copulas*. Springer Science & Business Media, 2007.
13. Marozzi M. Multivariate multidistance tests for high-dimensional low sample size case-control studies. *Statistics in Medicine* 2015; 34(9): 1511–1526.
14. Davis CS. *Statistical methods for the analysis of repeated measurements*. Springer Science & Business Media, 2002.
15. Brunner E, Bathke AC and Placzek M. Estimation of box's ε for low-and high-dimensional repeated measures designs with unequal covariance matrices. *Biometrical Journal* 2012; 54(3): 301–316.
16. Cai T, Liu W and Xia Y. Two-sample test of high dimensional means under dependence. *Journal of the Royal Statistical Society: Series B (Statistical Methodology)* 2014; 76(2): 349–372.
17. Gregory KB, Carroll RJ, Baladandayuthapani V et al. A two-sample test for equality of means in high dimension. *Journal of the American Statistical Association* 2015; 110(510): 837–849.
18. Jordan W, Tumani H, Cohrs S et al. Prostaglandin d synthase (β-trace) in healthy human sleep. *Sleep* 2004; 27(5): 867–874.
19. Brunner E, Domhof S and Langer F. *Nonparametric Analysis of Longitudinal Data in Factorial Experiments*. New York, USA: Wiley, 2002.
